# Neuroligin-1 mediates presynaptic maturation through brain-derived neurotrophic factor signaling

**DOI:** 10.1186/s12915-021-01145-7

**Published:** 2021-09-27

**Authors:** Andonia Petkova-Tuffy, Nina Gödecke, Julio Viotti, Martin Korte, Thomas Dresbach

**Affiliations:** 1 Institute for Anatomy and Embryology, University Medical Center Göttingen, Kreuzbergring 36, 37075 Göttingen, Germany; 2 Zoological Institute, Division of Cellular Neurobiology, TU Braunschweig, Spielmannstr. 7, 38106 Braunschweig, Germany; 3 Helmholtz Centre for Infection Research, Research group Neuroinflammation and Neurodegeneration, Imhoffenstr. 7, 38104 Braunschweig, Germany

**Keywords:** Neuroligin-1,Brain-derived neurotrophic factor, Presynaptic maturation

## Abstract

**Background:**

Maturation is a process that allows synapses to acquire full functionality, optimizing their activity to diverse neural circuits, and defects in synaptic maturation may contribute to neurodevelopmental disorders. Neuroligin-1 (NL1) is a postsynaptic cell adhesion molecule essential for synapse maturation, a role typically attributed to binding to pre-synaptic ligands, the neurexins. However, the pathways underlying the action of NL1 in synaptic maturation are incompletely understood, and some of its previously observed effects seem reminiscent of those described for the neurotrophin brain-derived neurotrophic factor (BDNF). Here, we show that maturational increases in active zone stability and synaptic vesicle recycling rely on the joint action of NL1 and brain-derived neurotrophic factor (BDNF).

**Results:**

Applying BDNF to hippocampal neurons in primary cultures or organotypical slice cultures mimicked the effects of overexpressing NL1 on both structural and functional maturation. Overexpressing a NL1 mutant deficient in neurexin binding still induced presynaptic maturation. Like NL1, BDNF increased synaptic vesicle recycling and the augmentation of transmitter release by phorbol esters, both hallmarks of presynaptic maturation. Mimicking the effects of NL1, BDNF also increased the half-life of the active zone marker bassoon at synapses, reflecting increased active zone stability. Overexpressing NL1 increased the expression and synaptic accumulation of BDNF. Inhibiting BDNF signaling pharmacologically or genetically prevented the effects of NL1 on presynaptic maturation. Applying BDNF to NL1-knockout mouse cultures rescued defective presynaptic maturation, indicating that BDNF acts downstream of NL1 and can restore presynaptic maturation at late stages of network development.

**Conclusions:**

Our data introduce BDNF as a novel and essential component in a transsynaptic pathway linking NL1-mediated cell adhesion, neurotrophin action, and presynaptic maturation. Our findings connect synaptic cell adhesion and neurotrophin signaling and may provide a therapeutic approach to neurodevelopmental disorders by targeting synapse maturation.

**Supplementary Information:**

The online version contains supplementary material available at 10.1186/s12915-021-01145-7.

## Background

Synaptic maturation is a complex event that turns newly formed synapses into fully functional units. Maturation endows synapses with adequate levels of stability, strength, and plasticity, and it optimizes them for their specific roles in a certain network. Activity-dependent maturation shapes developing circuits, while impaired synapse maturation profoundly affects brain function, contributing to neurodevelopmental and neurodegenerative disorders [1–3].

Neuroligins are postsynaptic cell adhesion molecules that regulate the number and maturation of synapses. Both loss and gain of function of neuroligins have been linked to the etiology of autism spectrum disorders (ASD) [4] and Alzheimer’s disease [5–7], highlighting their importance for brain function. There are four isoforms in rodents (NL1, NL2, NL3, NL4) and five in humans (NL1, NL2, NL3, NL4X, NL4Y), where NL1 (Neuroligin-1) is specific for excitatory synapses. Neuroligins bridge the synaptic cleft by binding to their presynaptic ligands, the neurexins. Increasing the levels of neuroligins increases the number of synapses on the dendrites of the overexpressing neurons, while decreasing their levels reduces the number of synapses [4, 8]. These effects require competition between cells, because they are only observed when neuroligins are overexpressed or knocked down in a small number of neurons among unperturbed neurons [9–13].

In contrast, both global and cell-specific changes in the levels of neuroligins affect synaptic maturation, indicating that promoting maturation represents a fundamental and essential function of neuroligins. For example, global triple knockout (KO) of NL1, NL2, and NL3 perturbs synaptic maturation as evidenced by reduced GABA (*gamma-*aminobutyric acid)-receptor recruitment to inhibitory synapses. In addition, subsets of synapses are silent in these mice [14]. Global knockout of NL1 reduces the strength of glutamatergic synapses by reducing NMDA(N-Methyl-D-aspartate)-receptor recruitment, and overexpression of NL1 in acute slices increases NMDA-receptor recruitment, synaptic strength, and the stability of active excitatory synapses [15, 16]. Likewise, overexpression of NL1 in vivo increases the size of dendritic spine heads and the recruitment of pre- and postsynaptic proteins [17, 18].

How do neuroligins exert their effects on synaptic maturation? We previously used cultured hippocampal neurons to determine which aspects of synaptic maturation are regulated by NL1 [19]. Overexpressing NL1 increased NMDA-receptor recruitment, presynaptic release probability, the number of presynaptic terminals with functional active zones (AZs), the number of recycling synaptic vesicles per terminal, and the stability of AZs in the absence of F-actin, all of which are hallmarks of maturation. These effects are reminiscent of the effects described for brain-derived neurotrophic factor (BDNF) in the context of neuronal development and plasticity. BDNF is a secreted transsynaptic signaling molecule of the neurotrophin family that binds to the TrkB (tyrosine receptor kinase B) neurotrophin receptor and the pan-neurotrophin receptor p75^NTR^. In addition to its functions in promoting neuronal survival, neurite differentiation, and axon guidance, BDNF mediates synaptic plasticity during postnatal development and in the mature nervous system [20, 21]. At the subcellular level, BDNF increases the number of synapses [22–24] and enhances synaptic protein recruitment, vesicle recycling, and release probability [25–28]. We hypothesize that this potency of BDNF may mediate AZ maturation and interact with neuroligin-based cell adhesion during network development.

Here, employing assays to test for structural and functional maturation, we found that applying BDNF to immature cultured neurons mimics the effects of overexpressing NL1 on synapse number and maturation. Moreover, BDNF application rescued the maturational defects of the loss of NL1. Conversely, overexpressing NL1 failed to rescue defective maturation in BDNF-depleted cultures. In fact, the action of NL1 on presynaptic maturation was severely impaired, suggesting that NL1 and BDNF act in concert to mediate presynaptic maturation.

## Results

### BDNF application mimics the positive effects of NL1 on structural and functional presynaptic maturation

In cultured neurons, presynaptic maturation can be readily monitored using two time windows: before DIV7 (day in vitro 7), nerve terminals are largely immature, and after DIV14, the majority of synapses have acquired a mature stage. During structural maturation, terminals become independent of F-actin, presumably by forming increasingly tight molecular complexes. The F-actin-disrupting drug latrunculin A (LatA) causes the loss of synaptic proteins from presynaptic terminals in young cultures but not in advanced cultures, providing a means to assay structural maturation [19, 29]. During functional maturation, the total recycling pool of synaptic vesicles increases. Functional maturation can be readily determined using synaptic vesicle (SV) recycling assays [19, 30]. Taking advantage of these two time windows, we applied gain-of-function assays before DIV7 (asking what enhances and accelerates maturation) and loss-of-function assays after DIV14 (asking what prevents maturation).

**Fig. 1.**
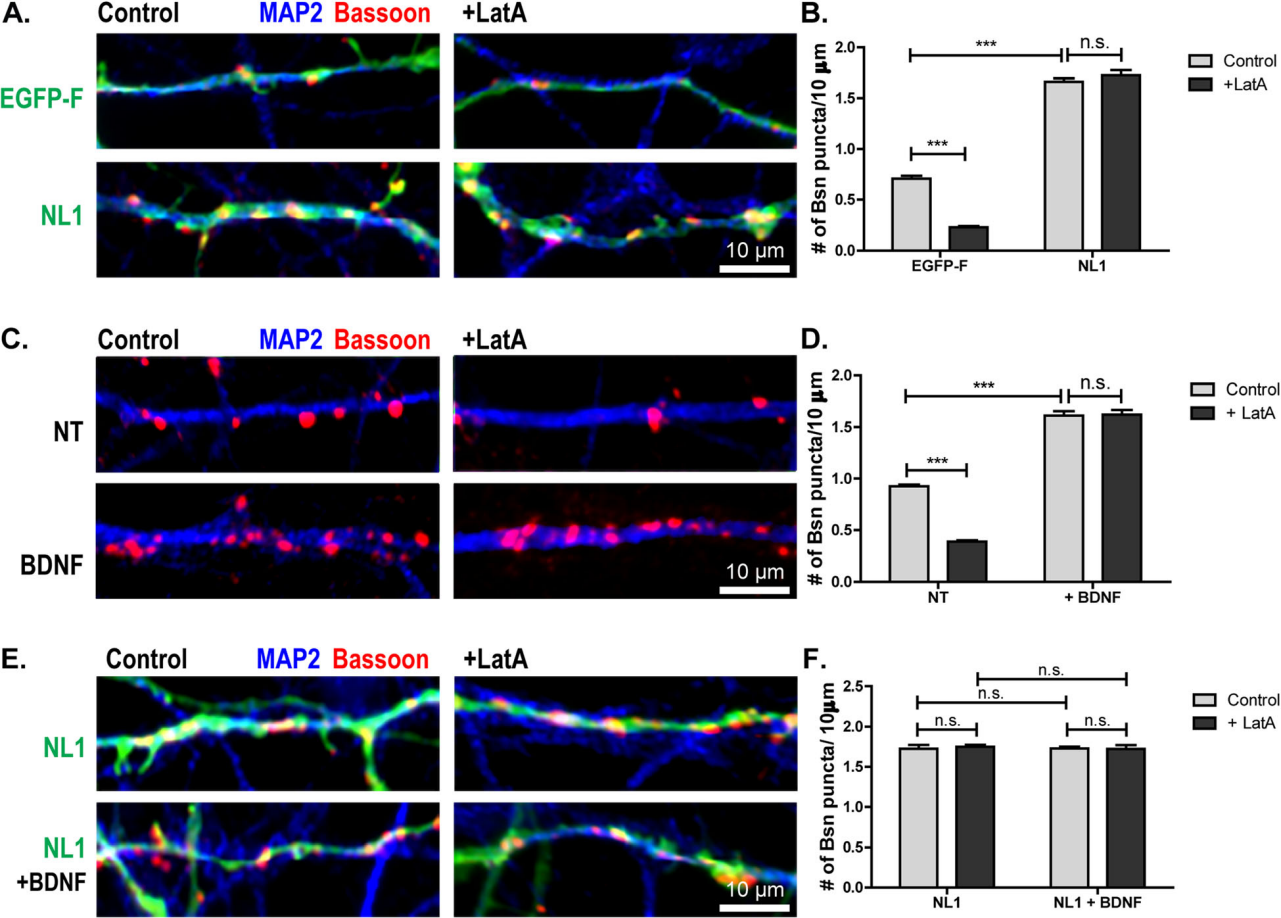
Induction of structural and functional AZ maturation by NL1 and BDNF. **A**, **B** Representative images and quantification of the number of bassoon puncta per 10 μm dendrite on DIV5 cultured hippocampal neurons transfected with membrane-targeted EGFP (farnesylated EGFP, EGFP-F) or GFP-tagged NL1, with and without prior LatA treatment and immunostained for bassoon (magenta). For separation of the three fluorescent channels, see Additional File 1: Fig. S1 A. Virtually all bassoon puncta formed on NL1-expressing cells are LatA resistant. Mean + SEM; *N*=3 experiments, *n*=10 cells per condition and experiment; two-way ANOVA with post hoc Sidak tests: interaction is significant, ***p< 0.0001, *F*(1, 116)=67.49; ***p< 0.0001 for GFP control vs. GFP + LatA, ***p< 0.0001 for GFP control vs. NL1 control, *p*> 0.05 for NL1 control vs. NL1 + LatA. **C**, **D** Representative images and quantification of the number of bassoon puncta per 10 μm dendrite on cultured hippocampal neurons treated with BDNF or buffer (not treated, NT) on DIV4, with and without LatA treatment, and fixed on DIV5 and immunostained for bassoon (magenta). Mean + SEM; *N*=3 experiments, *n*=10 cells per condition and experiment; two-way ANOVA with post hoc Sidak tests: interaction is significant, ***p< 0.001, *F*(1, 116)=67.49; ***p< 0.0001 for NT control vs. NT + LatA, ***p< 0.0001 for GFP control vs. NL1 control, *p*> 0.05 for NL1 control vs. NL1 + LatA. **E**, **F** Overexpression of NL1 together with a BDNF treatment does not lead to an additive effect. Representative images and quantification of the number of bassoon puncta per 10 μm dendrite on DIV5 DIV5 cultured hippocampal neurons transfected with NL1, treated with BDNF or buffer, treated with LatA or DMSO, and immunostained for bassoon (red) and MAP2 (blue). For separation of the three fluorescent channels, see Additional File 1: Fig. S1 B. Mean + SEM; *N*=3 experiments, *n*=10 cells per experiment and condition; two-way ANOVA with post hoc Sidak tests: *p*> 0.05 for all comparisons. Scale bar is 10 μm

To compare the potencies of NL1 and BDNF, we first confirmed our previous findings that NL1 mediates structural presynaptic maturation [19]. To this end, we transfected rat cultured neurons with NL1-GFP or membrane-targeted EGFP (farnesylated enhanced green fluorescent protein, EGFP-F) on DIV2. On DIV5 we fixed them with and without prior LatA treatment and immunostained them for bassoon to determine the number of AZs [19]. Overexpression of NL1 increased the number of bassoon puncta per 10 μm dendrite, indicating that it induced synapse formation (Fig. 1A; Additional File 1: Fig. S1). Virtually all bassoon puncta formed on NL1-overexpressing cells were LatA-resistant (Fig. 1B). As expected, the majority of bassoon puncta formed on EGFP-expressing cells were disassembled by LatA. In contrast, virtually all bassoon puncta formed on NL1-overexpressing cells were LatA-resistant, indicating that they had reached the advanced structural maturation state characteristic of DIV15 neurons (Fig. 1B). To test if BDNF also promotes presynaptic terminal maturation, we applied BDNF to cultured neurons on DIV4 and determined the LatA resistance of bassoon on DIV5. BDNF application increased the number of bassoon puncta, suggesting that it caused the formation of AZs (Fig. 1C, D). Virtually all bassoon puncta on BDNF-treated dendrites were LatA-resistant, reflecting advanced structural maturation. Thus, BDNF application and overexpression of NL1 have similar effects in inducing structural maturation of AZs (Fig. 1A–D). To assess whether the effects of NL1 overexpression and BDNF application are additive, we transfected cultured hippocampal neurons with NL1-GFP on DIV2 and treated them with BDNF or buffer on DIV4. On DIV5 we fixed them with and without prior LatA treatment and immunostained them for bassoon to determine the number of AZs. There was no significant difference between the conditions (Fig. 1E–F; Additional File 1: Fig. S1), indicating that the actions of NL1 and BDNF are not additive.

**Fig. 2.**
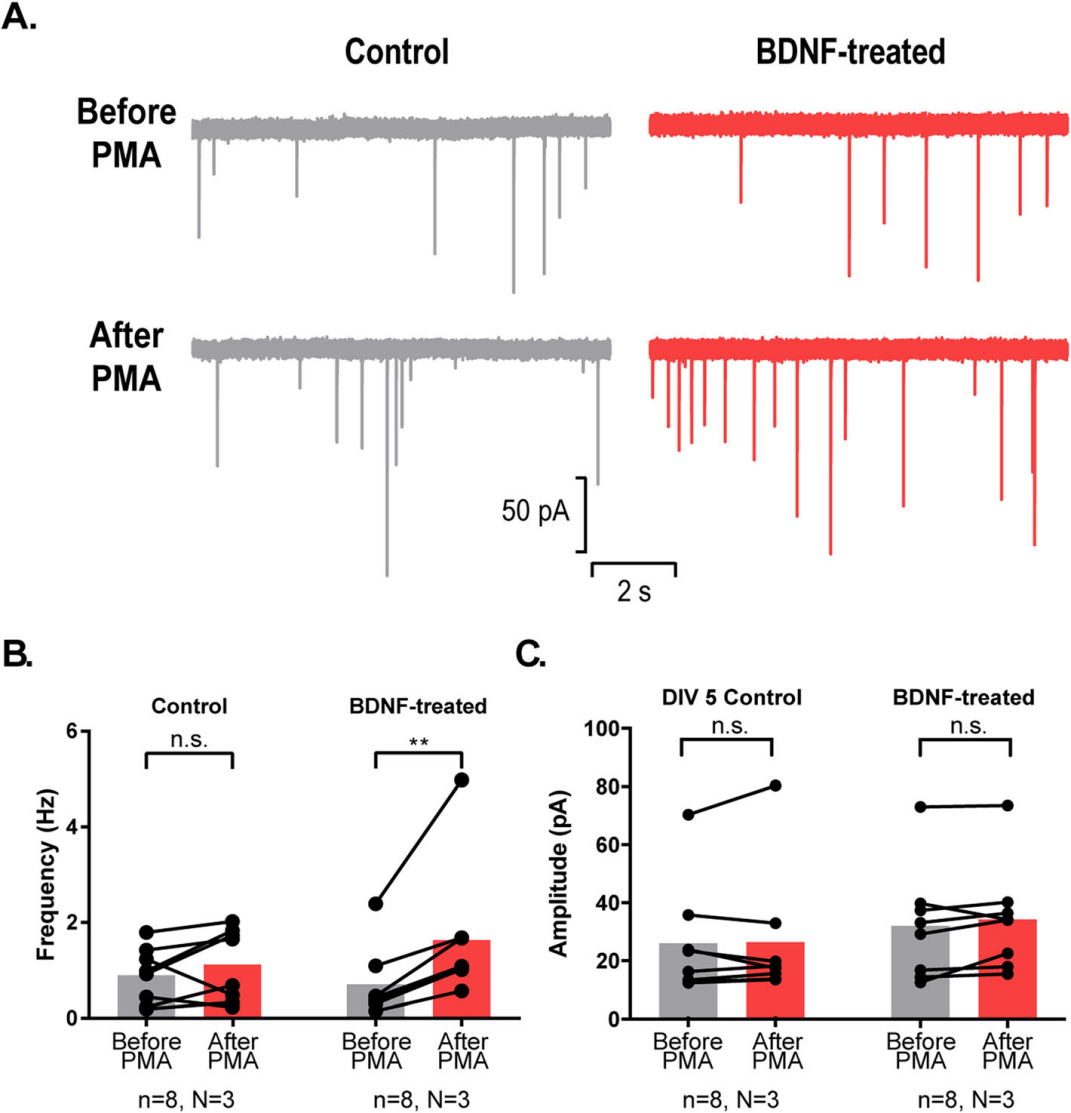
BDNF treatment makes synapses of DIV5 cultured neurons responsive to PMA. **A** Example traces of electrophysiological recordings showing miniature PSCs before and after application of PMA. **B** Frequency of miniature EPSCs is increased after application of PMA in BDNF-treated neurons but not in control neurons (*n* = number of cells; *N* = number of cultures; n.s.: non-significant change, **P=0.0078, Wilcoxon matched-pairs signed ranked test). **C** Amplitude of minature EPSCs is not changed by application of PMA in BDNF-treated neurons nor in control neurons (*n* = number of cells; *N* = number of cultures; n.s.: non-significant change, Wilcoxon matched-pairs signed ranked test)

In cultured neurons spontaneous synaptic transmission becomes responsive to the phorbol ester PMA (phorbol 12-myristate 13-acetate) upon reaching functional maturation. In particular, the frequency of miniature EPSCs (excitatory postsynaptic currents) is increased by PMA at DIV15, but not at DIV5 [31]. However, when NL1 is overexpressed at DIV5, PMA does increase miniature EPSC frequency (without affecting their amplitude), indicating that NL1 promotes this aspect of presynaptic maturation [19]. To test if the BDNF application has the same potency, we recorded miniature EPSCs before and after application of PMA. The frequency of miniature EPSCs (but not their amplitude) increased after PMA application in BDNF-treated neurons but not in control neurons (Fig. 2). Thus, BDNF application mimics the established effects of NL1 on structural and functional presynaptic maturation.

### BDNF application increases the lifetime of synapses in dissociated neurons and organotypic cultures

**Fig. 3.**
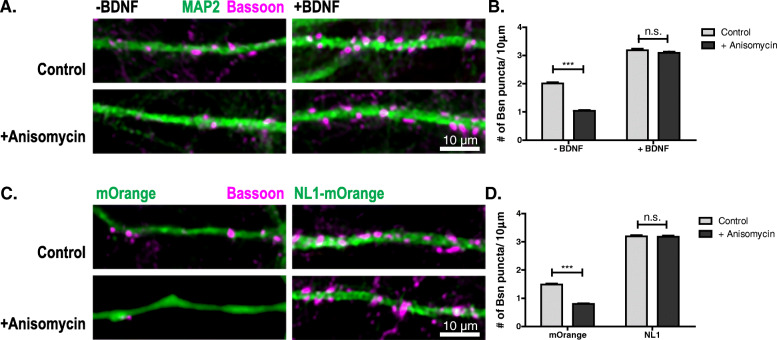
BDNF and NL1 increase the lifetime of AZs. **A** Cultured hippocampal neurons treated with buffer (-BDNF) or BDNF (+BDNF) on DIV4, treated with DMSO (control) or Anisomycin (+Anisomycin) on DIV5, fixed 6 h later and immunostained for bassoon (magenta) and MAP2 (green). **B** Quantification of the number of bassoon puncta per 10 μm dendrite for the conditions indicated in panel **A**. Mean + SEM; *N* = 3 experiments, *n* = 10 cells per experiment and condition; two-way ANOVA with post hoc Sidak tests: interaction is significant ***p=0.0001, *F*(1, 116)=99.82; ***p< 0.0001 for NT control vs. NT + Anisomycin; *p*> 0.05 for BDNF control vs. BNDF + Anisomycin. **C** DIV5 cultured hippocampal neurons transfected with mOrange or mOrange-tagged NL1 (green), treated with Anysomicin or DMSO for 6 h, fixed and immunostained for bassoon (magenta). **D** Quantification of the number of bassoon puncta per 10 μm dendrite for the conditions indicated in panel **C**. Mean + SEM; *N* = 3 experiments, *n* = 10 cells per experiment and condition; two-way ANOVA with post hoc Sidak tests: interaction is significant (***p< 0.0001, *F*(1, 116)=82.33; ***p< 0.0001 for mOrange control vs. mOrange + Anisomycin; *p*> 0.05 for NL1 control vs. NL1 + Anisomycin). Scale bar is 10 μm

**Fig. 4.**
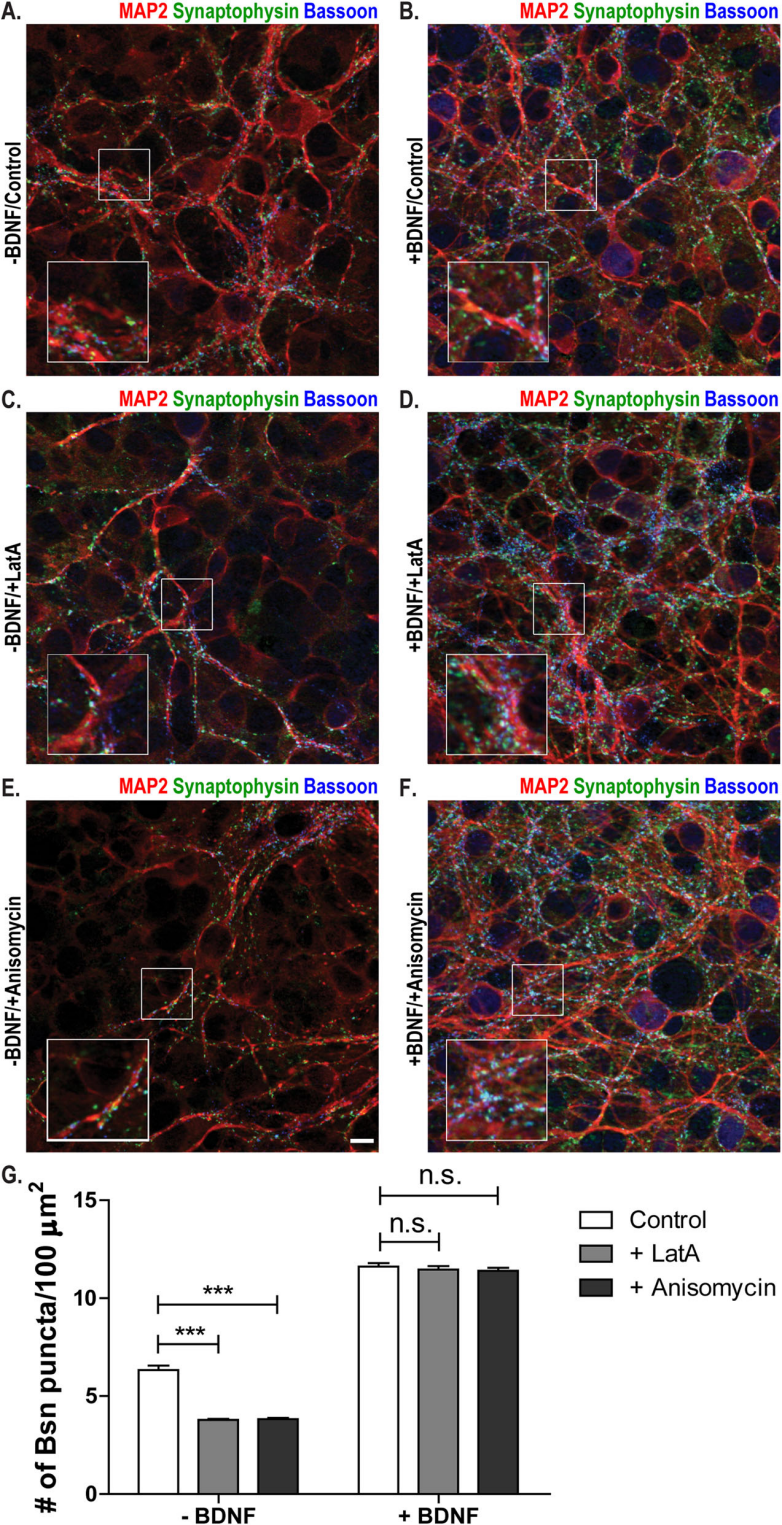
BDNF increases the structural maturation and the lifetime of AZs in organotypic cultures. **A** Hippocampal organotypic cultures treated with buffer (-BDNF) on DIV5; treated with DMSO (control) on DIV6; fixed 6 h later; and immunostained for MAP2 (red), synaptophysin (green), and bassoon (blue). **B** Hippocampal organotypic cultures treated with BDNF (+BDNF) on DIV5; treated with DMSO (control) on DIV6; fixed 6 h later; and immunostained for MAP2 (red), synaptophysin (green), and bassoon (blue). **C** Hippocampal organotypic cultures treated with buffer (-BDNF) on DIV5; treated with latrunculin A (+LatA) on DIV6; fixed 6 h later; and immunostained for MAP2 (red), synaptophysin (green), and bassoon (blue). **D** Hippocampal organotypic cultures treated with BDNF (+BDNF) on DIV5; treated with latrunculin A (+LatA) on DIV6; fixed 6 h later; and immunostained for MAP2 (red), synaptophysin (green), and bassoon (blue). **E** Hippocampal organotypic cultures treated with buffer (-BDNF) on DIV5; treated with anysomycin (+Anisomycin) on DIV6; fixed 6 h later; and immunostained for MAP2 (red), synaptophysin (green), and bassoon (blue). **F** Hippocampal organotypic cultures treated with BDNF (+BDNF) on DIV5; treated with anysomycin (+Anisomycin) on DIV6; fixed 6 h later; and immunostained for MAP2 (red), synaptophysin (green), and bassoon (blue). **G** Quantification of the number of bassoon puncta per 100 μm^2^ for the conditions indicated in panels **A**–**F**. Mean + SEM are shown; *N* = 3 experiments, *n* = 10 images per condition and experiment; two-way ANOVA with post hoc Sidak tests: interaction is significant (***p< 0.0001, *F*(2, 174)=38.43; ***p< 0.0001 for NT control vs. NT + LatA and for NT control vs. NT + Anisomycin; *p*> 0.05 for BDNF control vs. BDNF + LatA and for BDNF control vs. BDNF + Anisomycin). Scale bar is 10 μm

LatA resistance of synapses is a property of AZs that we and others have used to determine the developmental stage of synapses in cultured neurons, but which biologically relevant feature it represents has been unknown. We wondered whether LatA resistance might be a manifestation of increased lifetime, i.e., reduced turnover, of AZs. To test this, we blocked protein translation in cultured neurons using anisomycin. This assay has been used by Bednarek and Caroni [32] to determine the lifetime of bassoon at AZs. While anisomycin treatment reduced the number of bassoon puncta in immature control cultures, it did not lead to a reduction of bassoon puncta after BDNF application or NL1 overexpression (Fig. 3). We conclude that BDNF application and NL1 overexpression increase the lifetime of AZs. To test whether BDNF causes the same effect in a network with more preserved connectivity, we applied BDNF to immature organotypic cultures from the hippocampus. Indeed, BDNF application induced the LatA resistance of bassoon puncta and increased their lifetime in these slice cultures, too (Fig. 4).

### BDNF signaling participates in NL1-induced structural and functional presynaptic maturation

**Fig. 5.**
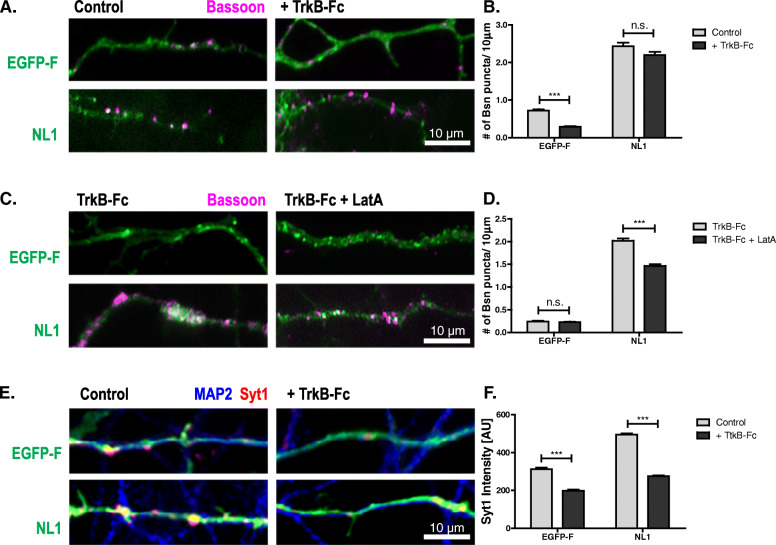
BDNF signaling participates in NL1 action. **A**, **B** DIV5 cultured hippocampal neurons transfected with farnesylated EGFP (EGFP-F) or NL1-EGFP (NL1) (green) grown in the absence or presence of TrkB-Fc and immunostained for bassoon (magenta), to determine the number of AZs. Mean + SEM are shown; *N* = 3 experiments, *n* = 10 cells per condition and experiment; two-way ANOVA with post hoc Sidak tests: effects of TrkB-Fc treatment (***p< 0.0001, *F*(1, 116)=24.09) and construct used (***p< 0.0001, *F*(1, 116)=708.9) are significant; ***p< 0.0001 for GFP control vs. GFP + TrkB-Fc; *p*> 0.05 for NL1 control vs. NL1 + TrkB-Fc. **C**, **D** TrkB-Fc-treated cultures with and without LatA treatment immunostained for bassoon, to determine the number of LatA-resistant AZs. Mean + SEM are shown; *N* = 3 experiments, *n* = 10 cells per condition and experiment; two-way ANOVA with post hoc Sidak tests: interaction is significant (***p< 0.0001, *F*(1, 116)=70,19); ***p< 0.0001 for NL1 + TrkB-Fc vs. NL1 + TrkB-Fc + LatA; *p*> 0.05 for GFP + TrkB-Fc vs. GFP + TrkB-Fc + LatA. **E**, **F** BDNF signaling is required for NL1-mediated functional presynaptic maturation. Representative images and quantification of cultured hippocampal neurons transfected on DIV2 with EGFP-F or NL1-EGFP (NL1) (green), grown in the absence or presence of TrkB-Fc, and stimulated in the presence of antibodies directed against the lumenal domain of synaptotagmin-1 (Syt1) to label recycling synaptic vesicles. After washing, the cells were fixed and immunolabeled with secondary antibodies against the Syt1 antibody (red) and with MAP2 antibodies (blue). For separation of the three fluorescent channels, see Additional File 2: Fig. S2. Mean ± SEM; *N* = 3 experiments, *n* = 10 cells per condition and experiment; two-way ANOVA with post hoc Sidak tests: interaction is significant (***p< 0.0001, *F*(1, 116)=66.60); ***p< 0.0001 for GFP control vs. GFP + TrkB-Fc and for NL1 control vs. NL1 + TrkB-Fc. Scale bar is 10 μm

To test if neuroligins require BDNF signaling to promote AZ maturation, we overexpressed NL1 in rat cultured neurons in the presence of TrkB-Fc, a soluble fragment of the BDNF receptor which is known to bind and scavenge secreted BDNF, thus reducing the concentration of BDNF released into the media [33]. TrkB-Fc reduced the number of bassoon puncta on GFP-expressing cells but did not block the action of NL1 in increasing puncta number (Fig. 5A, B). To test whether the bassoon puncta formed under conditions of perturbed BDNF signaling represent structurally mature or immature AZs, we determined the LatA resistance of bassoon in TrkB-Fc-treated cultures. TrkB-Fc clearly reduced the number of LatA-resistant bassoon puncta on NL1-expressing cells (Fig. 5C, D), indicating that a significant proportion of AZs formed by NL1 remain structurally immature when BDNF signaling is perturbed.

To test if BDNF signaling is required for functional maturation of NL1-induced synapses, we assayed depolarization-driven uptake of anti-synaptotagmin-1 antibodies. This assay exploits the fact that neurotransmitter release represents a cycle of exo- and endocytosis: upon exocytosis, the lumenal domains of synaptic vesicle transmembrane proteins become exposed at the plasma membrane surface of presynaptic nerve terminals. Bath-applied antibodies that detect such lumenal domains are taken up into the nerve terminal during the endocytotic phase of neurotransmitter release and can be detected by fluorophore-coupled secondary antibodies. Thus, the fluorescence intensity is proportional to the number of antibodies taken up during the stimulus and therefore reflects the number of vesicles undergoing the exo-endocytotic cycle. On DIV5, EGFP-F- or NL1–EGFP-expressing cultures were stimulated in the presence of antibodies directed against the lumenal domain of synaptotagmin-1 (Syt1), to label recycling synaptic vesicles. After washing, the cells were fixed and immunolabeled with secondary antibodies against the Syt1 antibody. NL1 increased the intensity of the Syt1 signal per terminal, indicating enhanced synaptic vesicle recycling in nerve terminals formed on NL1-expressing cells (Fig. 5E, F; Additional File 2: Fig. S2). TrkB-Fc reduced Syt1 immunofluorescence in terminals in control cultures, and it abolished the NL1-induced increase in synaptic vesicle recycling.

### BDNF is required for presynaptic maturation

**Fig. 6.**
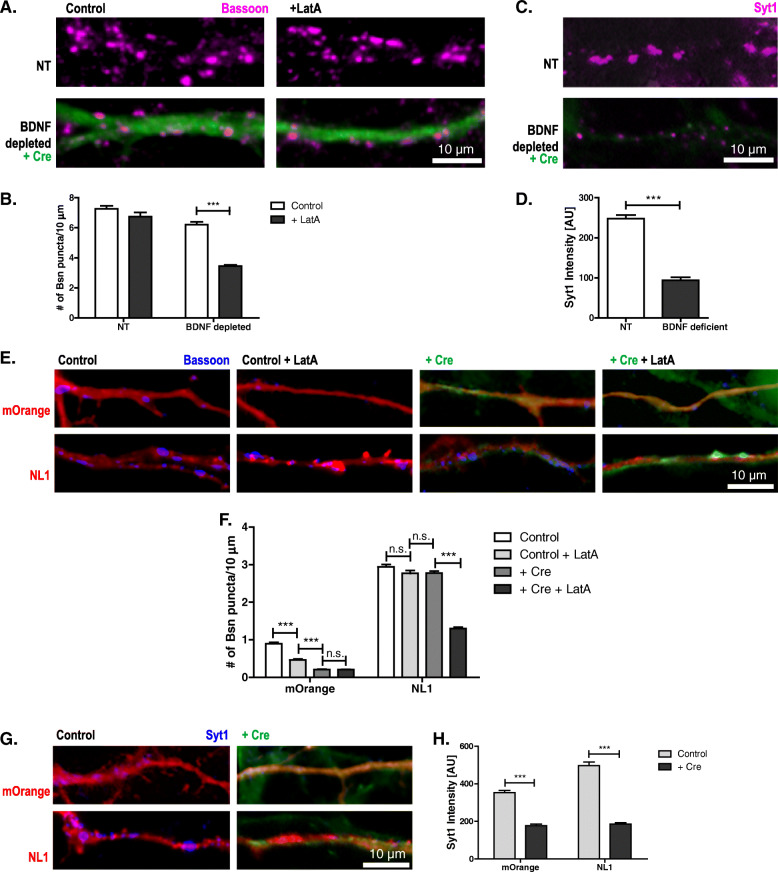
BDNF is required for endogenous and NL1-induced presynaptic maturation. Cultures of BDNF^lox/lox^ hippocampal neurons with Cre-recombinase viral transduction (green) to reduce BDNF levels compared to non-transduced cultures. **A**, **B** Representative images and quantification of the number of bassoon puncta per 10 μm dendrite on on DIV15 neurons, with and without prior 18-h LatA treatment, immunostained for bassoon (magenta) to determine the number of AZs. Mean + SEM; *N*=3 experiments, *n*=10 cells per condition and experiment; two-way ANOVA with post hoc Sidak tests: interaction is significant (***p< 0.0001, *F*(1, 116)=30.82); ***p< 0.0001 for BDNF depleted vs. BDNF depleted + LatA; *p*> 0.05 for NT vs. NT + LatA. **C** Representative images of cultures of BDNF^lox/lox^ hippocampal neurons with Cre-recombinase viral transduction (green) to reduce BDNF levels compared to non-transduced cultures, stimulated in the presence of antibodies directed against the lumenal domain of Syt1 to label recycling synaptic vesicles. After washing, the cells were fixed and immunolabeled with secondary antibodies against the Syt1 antibody (magenta). **D** Quantification of the fluorescence intensity of the Syt1 label. Mean + SEM; *N*=3 experiments, *n*=10 cells per condition and experiment; Student’s *t* test: ***p< 0.0001. **E**, **F** Representative images and quantification of the number of bassoon puncta per 10 μm dendrite on DIV6 BDNF^lox/lox^ cultures and Cre-recombinase transduced cultures transfected with mOrange or NL1-mOrange (NL1) (red) and immunostained for bassoon (blue). For separation of the three fluorescent channels, see Additional File 3: Fig. S3 A. Mean + SEM; *N*=3 experiments, *n*=10 cells per condition and experiment; two-way ANOVA with post-hoc Sidak tests: interaction is significant: ***p< 0.0001, *F*(3, 232)=98.00; ***p< 0.0001 for mOrange control vs. mOrange control + LatA, for mOrange control/control + LatA vs. mOrange + Cre and for NL1 + Cre vs. NL1 + Cre + LatA; *p*> 0.05 for mOrange + Cre vs. mOrange + Cre + LatA, for NL1 control vs. NL1 control + LatA, and for NL1 control/control + LatA vs. NL1 + Cre. **G, H** Representative images and quantification of cultured hippocampal neurons from BDNF^lox/lox^ mice with or without transduction with Cre-recombinase (green) transfected with mOrange or NL1–IRES–mOrange (NL1) (red) on DIV4. On DIV6 cultures were stimulated in the presence of antibodies against the lumenal domain of Syt1 to label recycling synaptic vesicles. After washing, the cells were fixed and immunolabeled with secondary antibodies against the Syt1 antibody (blue). For separation of the three fluorescent channels, see Additional File 3: Fig. S3 B. Mean ± SEM; *N*=3 experiments, *n*=10 cells per condition and experiment; two-way ANOVA with post-hoc Sidak tests: interaction is significant: ***p< 0.0001, *F*(1, 116)=29.33; ***p< 0.0001 for mOrange vs. mOrange + Cre and for NL1 vs. NL1 + Cre. Scale bar is 10 μm

To further investigate how BDNF affects NL1-induced presynaptic maturation, we tested DIV15 cultures from mice carrying two floxed *bdnf* alleles, where the BDNF gene was excised by transducing the cultures with *Cre*-recombinase-expressing lentivirus on the day of plating. In cultures from NL1-knockout mice presynaptic terminals fail to mature [19]. If BDNF is part of the underlying pathway, BDNF-depleted cultures should have impaired presynaptic maturation, too. To test this prediction, we determined the LatA resistance of AZs in DIV15 BDNF-depleted cultures. LatA application significantly reduced the number of bassoon puncta in *Cre*-transduced cultures but not in control cultures (Fig. 6A, B).

To test the functional maturation of presynaptic terminals in DIV15 BDNF-depleted cultures, we applied the Syt1-uptake assay. The Syt1-label intensity in Cre-transduced cultures was significantly reduced compared to control cultures (Fig. 6C, D), indicating that functional presynaptic maturation was also reduced in BDNF-depleted cultures. Thus, knockout of BDNF impairs presynaptic maturation in a way similar to knockout of NL1.

### NL1 fails to induce presynaptic maturation in BDNF knockout cultures

To further corroborate the notion that NL-induced presynaptic maturation requires BDNF, we overexpressed NL1 in BDNF-depleted mouse cultures on DIV4 and assayed maturation on DIV6. We co-expressed untagged full-length NL1 with IRES (internal ribosomal entry site)-driven mOrange, to visualize transfected neurons in GFP-Cre-transduced cultures. Applying LatA to mouse cultures significantly reduced the number of bassoon puncta on mOrange-expressing neurons, whereas the number of puncta on NL1-overexpressing neurons remained virtually unchanged (Fig. 6E, F; Additional File 3: Fig. S3 A), as expected from our results obtained in rat cultures transfected with NL1–GFP constructs. Transducing with Cre to remove BDNF had the following effect on NL1 overexpression: BDNF depletion did not affect the number of bassoon puncta on NL1-overexpressing neurons. In contrast, LatA application led to a significant reduction in the number of bassoon puncta on NL1-overexpressing neurons, indicating that in BDNF-depleted cultures NL1 failed to induce structural maturation. We then tested synaptic vesicle recycling in DIV6 BDNF-depleted cultures transfected with NL1–mOrange (NL1) or mOrange on DIV4. Knockout of BDNF significantly decreased the intensity of the Syt1 label in presynaptic terminals compared to mOrange-expressing control cells, and it virtually prevented the NL1-induced increase in SV recycling (Fig. 6G, H; Additional File 3: Fig. S3 B).

### Is neurexin binding required for presynaptic maturation?

To examine this, we overexpressed NL1 and an NL1 variant that does not bind to neurexins [34] in young cultures. Both WT NL1 and the neurexin-binding-deficient mutant generated the same LatA resistance and Syt1 uptake levels when overexpressed on DIV5 in rat cultures (Additional File 4: Fig. S4). LatA resistance levels were also similar when we compared the action of both constructs in NL1-KO cultures (Additional File 5: Fig. S5). Thus, neurexin binding may be dispensible for these aspects of NL1-induced presynaptic maturation.

### BDNF rescues defective presynaptic maturation in NL1-KO cultures

**Fig. 7.**
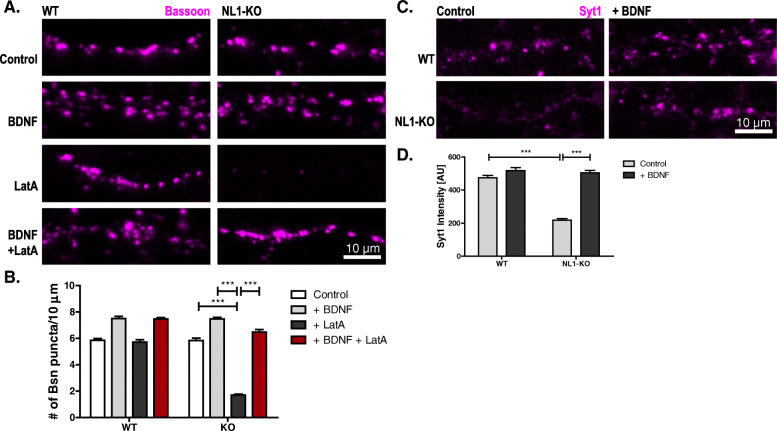
BDNF restores presynaptic maturation in NL1-deficient cultures. **A**, **B** Representative images and quantification of cultured cortical neurons from WT and NL1-KO mice fixed on DIV15. Prior to fixation, the neurons were treated with either BDNF for 22 h, LatA for 18 h, or both (see labeling on the left). Following fixation, the neurons were immunolabeled with a bassoon antibody (magenta). Mean + SEM; *N* = 3 experiments, *n* = 10 cells per condition and experiment; two-way ANOVA with post hoc Sidak tests: interaction is significant: ***p< 0.0001; *F*(3, 232)=95.61; ***p< 0.0001 for KO control vs. KO + LatA, for KO + BDNF vs. KO + LatA, and for KO control vs. KO + BDNF + LatA. Scale bar is 10 μm. **C**, **D** Representative images and quantification of cultured cortical neurons from WT and NL1-KO mice fixed on DIV15. On DIV15 cultures were stimulated in the presence of antibodies against the lumenal domain of synaptotagmin-1 (Syt1) to label the recycling of synaptic vesicles. After washing, the neurons were fixed and immunolabeled with secondary antibodies against the Syt-1 antibody (magenta). Mean ± SEM; *N* = 3 experiments, *n* = 10 cells per condition and experiment; two-way ANOVA with post hoc Sidak tests: interaction is significant: ***p< 0.0001; *F*(1, 116)=68.32; ***p< 0.0001 for WT control vs. KO control and for KO control vs. KO + BDNF. Scale bar is 10 μm

Why does NL1 fail to promote maturation in the absence of BDNF? One explanation is that NL1 elicits or enhances BDNF signaling to promote presynaptic maturation. In this case, exogenous BDNF should rescue maturation in NL1-KO cultures, which fail to mature [19]. To test this, we applied BDNF to advanced cortical cultures (DIV14) from WT (wild type) and NL1-KO mice and tested them for structural and functional maturation a day later. Remarkably, BDNF application reestablished structural and functional maturation in these NL1-KO cultures within one day (Fig. 7).

### NL1 increases postsynaptic BDNF levels to induce presynaptic maturation

**Fig. 8.**
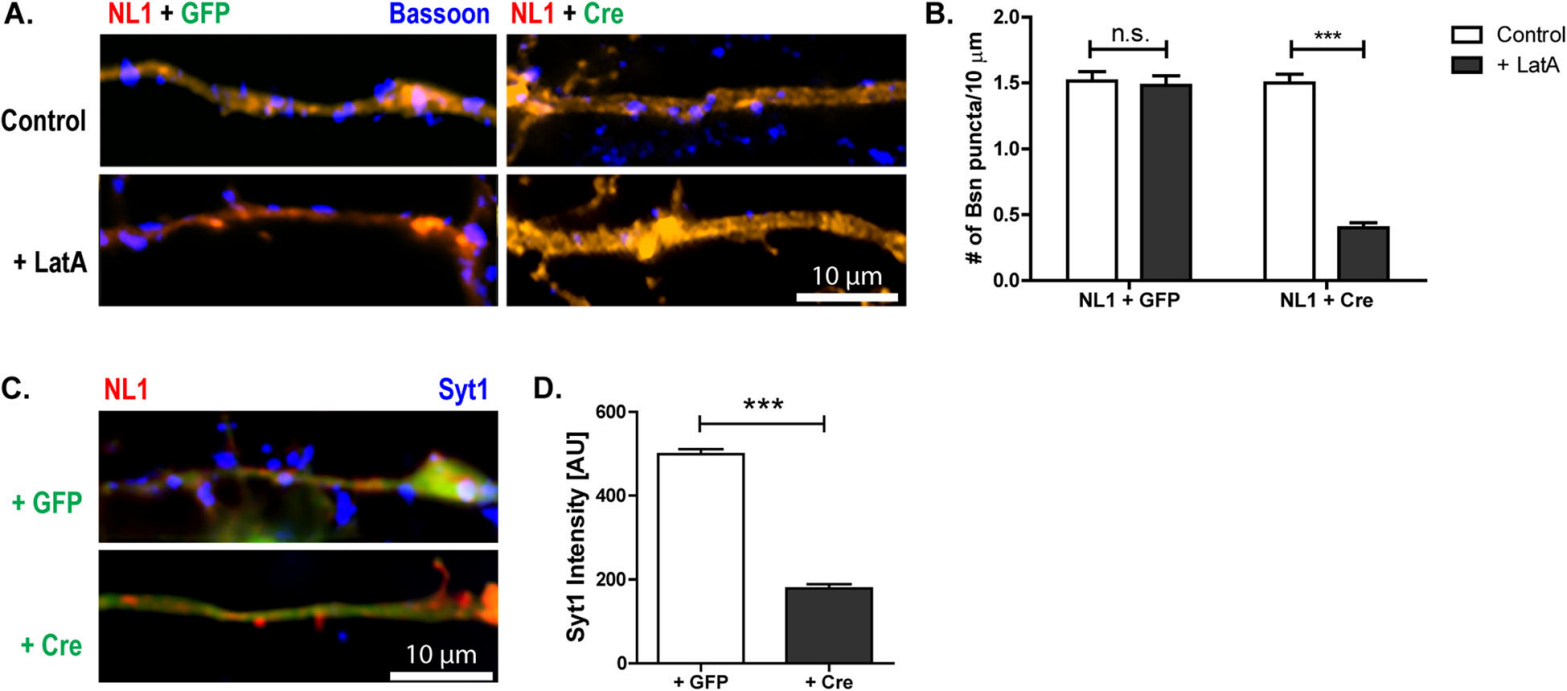
Postsynaptic BDNF is required for NL1-induced presynaptic maturation. **A**, **B** Representative images and quantification of cultured hippocampal neurons from BDNF^lox/lox^ mice co-transfected with NL1–IRES–mOrange (red) + Cre/GFP (green) on DIV4. Neurons were fixed on DIV6 and immunostained for bassoon (blue) following a 6-h LatA treatment. For separation of the three fluorescent channels, see Additional File 6: Fig. S6 A. Mean ± SEM; *N* = 3 experiments, *n* = 10 cells per condition and experiment; two-way ANOVA with post hoc Sidak tests: interaction is significant: ***p< 0.0001; *F*(1, 116)=259.8; ***p< 0.0001 for NL1 + Cre control vs. NL1 + Cre + LatA; *p*> 0.05 for NL1 + GFP control vs. NL1 + GFP + LatA. Scale bar is 10 μm. **C**, **D** Representative images and quantification of cultured hippocampal neurons from BDNF^lox/lox^ mice co-transfected with NL1-mOrange (red) + Cre/GFP (green) on DIV4. On DIV6 cultures were stimulated in the presence of antibodies against the lumenal domain of synaptotagmin-1 (Syt1) to label recycling synaptic vesicles. After washing, the cells were fixed and immunolabeled with secondary antibodies against the Syt1 antibody (blue). For separation of the three fluorescent channels, see Additional File 6: Fig. S6 B. Mean ± SEM; *N* = 3 experiments, *n* = 10 cells per condition and experiment; Student’s *t* test: ***p< 0.0001. Scale bar is 10 μm

To test whether the BDNF that mediates neuroligin-induced presynaptic maturation is released from the postsynaptic side, we introduced Cre recombinase and NL1 into a small number of neurons from *bdnf* ^lox/lox^ mice by transfection rather than viral transduction. Using this paradigm, only a small number of neurons (dozens per coverslip containing 70,000 cells) expressing NL1 also expressed Cre, thus removing BDNF from the postsynaptic cell. To verify co-transfection of NL1 and Cre, the NL1 construct co-expressed mOrange and the Cre plasmid co-expressed GFP from an internal ribosomal entry site. As a control, we co-transfected cells with NL1 and GFP. We then tested the structural and functional maturation of the boutons contacting these neurons using the LatA and Syt1-uptake assay (Fig. 8A–D; Additional File 6: Fig. S6 A-B). At dendrites of NL1-overexpressing neurons co-transfected with Cre, the number of LatA-resistant bassoon-stained puncta as well as the Syt-1 fluorescence intensity was significantly decreased compared to the neurons co-transfected with GFP as a control, indicating that postsynaptic BDNF is required for NL1-induced presynaptic maturation.

**Fig. 9.**
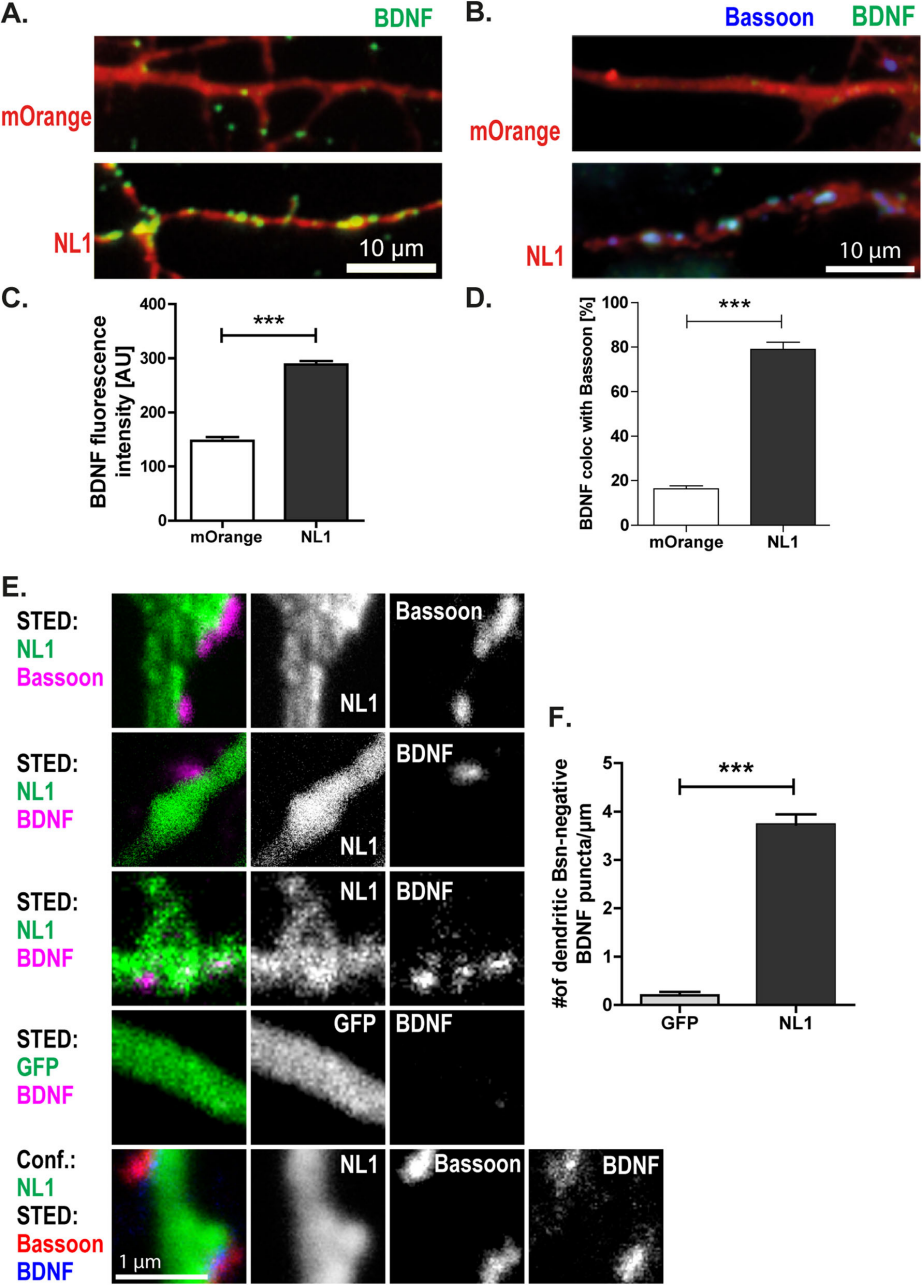
Effects of NL1 overexpression on BDNF fluorescence intensity and distribution. **A**, **B** Overexpressing NL1 increases BDNF immunofluorescence at AZs. Representative images and quantification of BDNF fluorescence intensity in the dendritic region of neurons expressing mOrange or NL1–IRES–mOrange. On DIV5 the neurons were fixed and immunostained for BDNF (green). The average intensity of fluorescent puncta was quantified. Mean ± SEM; *N* = 3 experiments, *n* = 10 cells per condition and experiment; Student’s *t* test: ***p< 0.0001. Scale bar is 10 μm. **C**, **D** BDNF immunosignals colocalize with bassoon in NL1 overexpressing neurons. Mean ± SEM; *N* = 3 experiments, *n* = 10 cells per condition and experiment; Student’s *t* test: ***p< 0.0001. Scale bar is 10 μm. **E**, **F** STED nanoscopy images showing the distribution of BDNF. **E** DIV5 cultured hippocampal neurons transfected with NL1-GFP or GFP and immunostained for GFP, bassoon, and BDNF (see labels). **F** Average number of dendritic BDNF puncta per 1 μm. Mean + SEM; *N* = 3 experiments, *n* = 10 cells per experiment and condition; Student’s *t* test: ***p< 0.0001. Scale bar is 1 μm

Activity-induced expression is a central feature of BDNF, and NL1 can enhance neuronal activity by enhancing NMDA-receptor activity. To test whether NL1 leads to an increased expression of BDNF, we transfected DIV3 cultured hippocampal neurons with NL1–IRES–mOrange or mOrange as a control. After fixation, we immunostained the neuronal cultures for BDNF. We observed a significant increase in BDNF fluorescence in and along dendrites of NL1-overexpressing neurons compared to control neurons transfected with mOrange (for mOrange = 144.81 ± 38.39; for NL1 = 292.54 ± 14.88; Fig. 9A, B). Some of these signals were relatively small and homogeneously distributed, while another group occurred as local accumulations. NL1 overexpression increased the colocalization of these BDNF accumulations with bassoon from 14.97 ± 1.93% to 79.22 ± 3.97% (Fig. 9C, D). Thus, NL1 increases BDNF expression and recruitment to synapses. In addition, we used dual-color STED (stimulated emission depletion) microscopy to further characterize the distribution of BDNF (Fig. 9E, F). At nanoscopic resolution, bassoon puncta were observed adjacent to NL1-GFP-positive dendrites, which is consistent with presynaptic localization of bassoon and postsynaptic membrane localization of NL1-GFP. In addition, we found two categories of BDNF signals. Some BDNF signals were distributed inside dendrites. Dendrites of GFP-expressing neurons had on average 0.20 ± 0.23 dendritic BDNF puncta per 1 μm, while dendrites of NL1-expressing neurons had on average 3.73 ± 1.26 of BDNF puncta. Thus, NL1 expression significantly increased the number of dendritic BDNF signals. The other category of BDNF signals colocalized with bassoon at sites adjacent to NL1-GFP-positive dendrites, suggesting presynaptic localization of these BDNF signals (Fig. 9E, F). In summary, NL affects the distribution of BDNF on both sides of the synapse: NL1 recruits BDNF to synapses, and these synaptic accumulations of BDNF appear to be primarily presynaptic. Strikingly, postsynaptic BDNF is increased by and required for the action of NL1.

Together, our data suggest that BDNF is an essential novel participant in NL1-mediated presynaptic maturation.

## Discussion

Synapses go through stages of maturation to create fully functional networks. Studying early network formation in cultured neurons, we found that presynaptic maturation requires the concerted action of neuroligin-1 and BDNF. BDNF application mimicked the boosting effects of neuroligins on AZ stability and synaptic vesicle recycling. Perturbing endogenous BDNF signaling reduced both ongoing maturation and the enhancing effects of NL1. BDNF appeared to act downstream of NL1, permitting exogenous BDNF to restore presynaptic maturation in NL1-knockout neurons. Our data reveal that BDNF and NL1 jointly mediate structural and functional presynaptic maturation, thus implicating neurotrophin action in neuroligin-based signaling.

### Presynaptic maturation: distinct vs. overlapping pathways

Three scenarios could account for this cooperative action between NL1 and BDNF. First, presynaptic maturation could be accomplished through the combined effects of two independent pathways: one involving NL1-based cell adhesion and one involving BDNF signaling, which add up to full maturation. This seems unlikely for functional maturation, because the effects of NL1 in enhancing SV recycling were virtually abolished when BDNF signaling was blocked. If the pathways were independent, the contribution of NL1 to mediating presynaptic maturation should be unaffected. Structural maturation was reduced by 44% in BDNF-depleted cultures and by 71% in NL1-deficient cultures at DIV15. If maturation was mediated by two independent pathways, the contributions of BDNF and NL1 should not exceed 100%. Note that we may underestimate the importance of BDNF for structural maturation, because the floxed BDNF cultures were rendered BDNF-depleted via viral delivery of Cre; hence, residual BDNF—produced by uninfected neurons or incomplete Cre action—may still exist in the media. Thus, even this cautious estimate argues against two independent pathways. This conclusion is supported by the fact that applying BDNF to NL1-overexpressing cells did not further enhance maturation, indicating that the effects of overexpressing NL1 and applying BDNF are not additive.

Second, a cell-adhesion-based pathway and a neurotrophin-based pathway each may have the potency to generate full maturation, albeit through separate molecular cascades. This could reflect a redundant scenario to ensure proper maturation, and the pathways could depend on each other such that one is upregulated when the other one lags behind. We cannot exclude this possibility, but our data show that this potency—if it existed—is not used during synaptogenesis, since the absence of NL1 or BDNF in knockouts is not compensated for by the presence of the respective other pathways.

Third, NL1 and BDNF may act in a shared pathway or in closely overlapping pathways. Three aspects argue for this scenario. First, the effects of NL1 and BDNF were not additive. Moreover, BDNF fully rescued impaired presynaptic maturation caused by the loss of NL1, but NL1 did not rescue impaired maturation caused by BDNF deficiency. Thus, NL1 and BDNF cannot be acting in separate pathways and instead must act in the same or in overlapping pathways. Furthermore, overexpression of NL1 increased BDNF expression in the transfected neurons, indicating that the action of NL1 affects BDNF, providing a hint at the site of overlap.

### The overall effects of BDNF and NL1

A joint action of BDNF and NL1 appears to be plausible based on our new observations and on the known actions of these molecules. BDNF application increased the number of bassoon puncta and AZ stability in young cultures, and BDNF deprivation prevented the acquisition of the mature state in advanced cultures. The effects of BDNF application and BDNF deprivation resemble the effects of NL1 overexpression and NL1 knockout, respectively [19], suggesting that the two molecules are acting together during synapse maturation. Several known properties of BDNF are reminiscent of the effects of NL1. For example, BDNF and NL1 increase the levels of SV proteins [17, 19, 25, 35]. BDNF and NL1 also increase release probability [19, 27]. These similarities between the proteins inspired us to study whether BDNF and NL1 act in concert to mediate presynaptic maturation.

BDNF increased the number of bassoon puncta in our young cultures. This is consistent with increased synapse numbers in advanced (DIV14) cultures and postnatal (P7) slice cultures treated with BDNF [22, 23]. BDNF did not increase the frequency of miniature EPSCs (minis). This suggests that the newly formed accumulations of Bassoon are located at nascent synapses that may be silent at this early stage of synaptogenesis. This is consistent with the increase in the number of bassoon puncta and lack of effect on mini frequency observed after NL1 overexpression at the same culture stage [19] and highlights the similarity of the effects of BDNF and NL1. In our study, we focus on the effects of NL1 and BDNF on the maturation of existing synapses.

The importance of BDNF for both NL1-induced and endogenous maturation of SV recycling is consistent with the known role of BDNF in increasing the number of docked SVs and the extent of SV recycling in advanced stages (≥ DIV 13) of culture development [26, 28] and in P7 slice cultures [25]. These effects of BDNF are usually interpreted as a modulatory element acting in existing networks. Our data indicate that this potency of BDNF is also a crucial component of NL1-mediated presynaptic maturation.

BDNF also mimicked and mediated—at least in part—the effects of NL1 on AZ stabilization after F-actin disruption by LatA. In addition, knockout of either BDNF or NL1 prevented the acquisition of the structurally mature state, as shown before for NL1 [36]. Moreover, BDNF mimicked the positive effect of NL1 on phorbol ester sensitivity of synaptic transmission, another hallmark of presynaptic maturation [19, 31]. Overall, our data show that BDNF is a necessary component for the NL1-mediated effects on structural and functional maturation**.** It is unlikely that BDNF acts as a generally permissive element, e.g., by promoting axonal outgrowth and dendritic arborization, because the number of AZs was not reduced in advanced BDNF-depleted cultures: like in NL1-KO cultures, presynaptic terminals formed and decorated dendrites at a normal density but remained at an immature state. We propose that NL1 harnesses the positive effects of BDNF signaling on presynaptic terminals to boost presynaptic maturation during the early stages of network formation.

Although LatA resistance of AZs is a hallmark of presynaptic maturation in cultured neurons [19, 29, 37], which aspect of synapse function the assay actually reports is unknown. Synapses from NL1 single knockouts fail to become LatA-resistant but display normal release probability, suggesting that LatA resistance does not correlate with synaptic strength [15, 19, 38]. We hypothesized that LatA resistance reflects certain aspects of AZ stability. Indeed, by blocking protein translation with anisomycin, we found that BDNF and NL1 each increased the half-life of bassoon in immature synapses. This reveals one mode by which BDNF and NL1 stabilize AZs. Using the anisomycin assay, Bednarek and Caroni [32] showed that learning induced by enriched environment involves temporarily increased turnover of bassoon. The NL1–BDNF interaction reported here may help stabilize such synapses after remodeling; conversely, dysfunctional NL1–BDNF signaling may cause ongoing remodeling. Interestingly, four mouse models of autism, including patDp/+, NLG3 R451C, BTBR, and Fmr1-KO mice, show increased protein turnover at spines as well as increased formation and elimination of spines [39–44]. The concerted action of BDNF and NL1 observed here may be a means to stabilize AZs during synapse maturation, to prevent ongoing remodeling.

### How do NL1 and BDNF interact?

We have previously shown that structural maturation requires both NL1 and NMDA-receptor activity, suggesting that neuronal activity constitutes an additional element in the process of synaptic maturation [36]. Notably, both NL1 and BDNF recruit NMDA receptors to synapses [9, 15, 16, 19, 45]. In our study, overexpression of NL1 increased the fluorescence intensity of BDNF immunosignals. NL1 might increase network activity by recruiting NMDA receptors, thereby allowing for activity-dependent BDNF expression and secretion. Subsequently, BDNF could further enhance NMDA-receptor activity, Ca^2+^ influx, and downstream signaling. Ca^2+^ signaling, via CaMKII (Ca^2+^/calmodulin-dependent protein kinase II), promotes NL1 surface expression and function, as well as BDNF expression and secretion [46–48]. Thus, NMDA-receptor–CaMKII signaling could link NL1 and BDNF action in presynaptic maturation.

In which sequence do NL1 and BDNF act? While BDNF application rescued NL1 deficiency, NL1 overexpression failed to rescue BDNF deficiency. This indicates that BDNF is downstream of NL1 action. This is consistent with the upregulation of BDNF by NL1. Intriguingly, since neuronal activity is an element in the process, a circular system could arise, where BDNF and NL1 stimulate each other’s actions. In particular, NL1 could enhance NMDA-receptor-dependent neuronal activity and CaMKII signaling, thus increasing both BDNF expression and secretion. This in turn increases surface expression of NMDA receptors, thus closing the feedback loop. In addition, the positive regulation of NL1 via CaMKII [46] further strengthens the loop.

In this scenario, NL1 is the only protein that is exclusively postsynaptic. Thus, such a loop could involve both sides of the synapse but is likely triggered postsynaptically by NL1. Whether BDNF is released from axons or dendrites has been controversial, because secretion from either side of the synapse has been observed. The pathways are probably complex and likely include contributions of both pre- and postsynaptic BDNF. Here, we found that presynaptic inputs induced by NL1 overexpression in BDNF-depleted neurons failed to mature. Thus, NL1 action relied on locally produced BDNF released from the transfected neuron. Of note, the results do not exclude additional effects by presynaptically released BDNF. While BDNF seems to be exclusively presynaptic in the hippocampal CA3 and CA1 of 8-week-old animals [49], our findings for NL1-dependent maturation are consistent with dendritic release in cultured neurons [50, 51] and—in the context of long-term synaptic plasticity—in intact brain tissue [52–54]. Our data revealed that NL1 induced presynaptic maturation via dendritic release of BDNF. In addition, NL1 recruited BDNF to synapses. This synaptically recruited BDNF colocalized with bassoon at the nanoscopic level, suggesting a presynaptic localization. This presynaptic BDNF may also contribute to synaptic maturation, or be recruited to equip the synapses for synaptic plasticity in mature networks.

### Potential presynaptic mechanisms

What are the presynaptic events that follow NL-induced BDNF signaling? Binding of BDNF to TrkB receptors is likely involved, because scavenging BDNF with soluble TrkB-Fc bodies impaired maturation. Interestingly, BDNF treatment increases F-actin polymerization via TrkB–LIMK1 (LIM domain kinase 1) interaction during axonal outgrowth [55], raising the possibility that acquisition of LatA resistance could be due to enhanced stabilization of F-actin.

The evidence that the architecture of synapses becomes independent of F-actin during development was originally found by Zhang and Benson [29] and later shown to involve N-Cadherin signaling [37]. This is remarkable, since N-Cadherin recruits NL1 to synapses [56], suggesting that N-Cadherin–NL1-based cell adhesion triggers BDNF-signaling to induce LatA resistance of AZs. The biological purpose of LatA resistance is unclear. Also, what replaces F-actin as a stabilizing compound during neuronal development is unclear. At least two principal mechanisms are conceivable: (a) the AZ could become stabilized by transsynaptic anchoring involving direct physical connections to the PSD; (b) AZ molecules could become interconnected, thus tightening the cytomatrix of the AZ inherently. Impairment of either of these mechanisms could account for the reduced LatA resistance as assayed in BDNF and NL1-KO cultures. For our gain-of-function experiments, a third option cannot be excluded: application of BDNF and overexpression of NL1 may increase the levels of F-actin, thus enhancing F-actin stabilization so that some of it survives the LatA treatment. Synaptic sites of extremely stable F-actin have been previously observed [29].

In either case, i.e., through increased F-actin levels or through increased inherent stability, NL1 and BDNF could make AZs independent of ongoing actin remodeling and thus allow for a simple mechanism to stabilize mature Azs while remodeling mechanisms continue. Conversely, before LatA resistance is established, i.e., in the structurally immature state, AZs may be particularly malleable. In cultures from NL1-KO mice, which have normal synapse numbers but fail to acquire LatA resistance, the tenacity of excitatory synapses is reduced. The turnover of synaptic proteins as well as the fluctuation of their concentration at synapses is increased, and synaptic activity destabilizes synapses in NL1-KO cultures [57]. The LatA-resistant state may thus protect synapses against activity-induced changes in mature cultures, and the LatA-sensitive state may allow for structural plasticity in early cultures and during conditions where synapses have to remodel.

### Outlook

Activity-dependent synapse maturation is a central event in network refinement, and failures in this process likely contribute to neurodevelopmental disorders such as ASD. Interestingly, many proteins implicated in ASD are regulated by neuronal activity, emphasizing the importance of understanding the underlying signaling pathways and identifying convergent pathways. Both NL1 and BDNF are regulated in an activity-dependent manner and initiate processes that further enhance neuronal and network activity [58]. Through their interaction in presynaptic maturation, a new site of convergence between two signaling systems emerges. This novel insight has concrete benefits: defects in both NL1 function and BDNF secretion have been implicated in ASD in humans and behavioral abnormalities in mice [4, 59, 60]. Thus, the potency of BDNF to overcome the lack of maturation caused by NL1 deficiency provides an opportunity for therapeutic strategies. This interplay might extend beyond synapse maturation, because NL1 also promotes the survival of newborn neurons in the adult hippocampus [11]. At the cell biological level, our data suggest a scenario where neuroligins interact with neurexins to promote synapse formation and subsequently switch to a different receptor or even act without a presynaptic receptor to induce BDNF release and synaptic maturation. The multiple roles of BDNF for brain development and plasticity require tight spatial and temporal control of BDNF expression and release. Neuroligins could provide this control in the context of presynaptic maturation, by linking cell adhesion and synaptic activity to local BDNF secretion. It will be interesting to see whether this transsynaptic teamwork between NL1 and BDNF is also effective during synapse maturation in the adult brain, e.g., during constitutive synapse turnover, plasticity-related synapse formation, and adult neurogenesis.

## Conclusions

To induce presynaptic maturation, NL1 relies on the presence and secretion of BDNF. In this emerging pathway NL1 increases the expression and synaptic recruitment of BDNF, and BDNF subsequently promotes structural and functional presynaptic maturation. Structural maturation involves increased lifetime of active zones. Acting downstream of NL1 in this pathway, BDNF can restore defective presynaptic maturation in NL1-knockout neurons at advanced stages of neuronal development. Thus, BDNF signaling is a novel and essential participant of an emerging transsynaptic pathway originating from NL1. The fact that BDNF can restore defective synapse maturation in NL1-deficient neurons is important for therapeutic concepts addressing reduced maturation in autism spectrum disorders.

## Methods

### Cell culture

Primary hippocampal neuron cultures were prepared from E19 Wistar rats, and primary cortical neuron cultures were prepared from P0 NL1-WT and NL1-KO mice essentially as described previously [19]. The hippocampal neurons were plated at a density of 55,000 cells/cm^2^ on polyethylenimine (PEI)-coated coverslips in 24-well plates. Twelve hours after plating, the original Dulbecco’s modified Eagle’s medium (DMEM) solution containing 10% FCS (fetal calf serum), 100 μg/mL penicillin, 100 μg/mL streptomycin, and 1% L-glutamine was exchanged for Neurobasal medium with the same additives and B27 supplement.

Primary hippocampal mouse cultures were prepared from C57BL/6 J-SV129 mice on E16.5. Immediately after being taken out, the hippocampi were placed in ice-cold GBSS (Gey’s balanced salt solution: 137 mM NaCl, 5.55 mM D-glucose, 4.89 mM KCl, 0.33 mM KH_2_PO_4_, 1.033 mM MgCl_2_·6H_2_O, 0.284 mM MgSO_4_·7H_2_O, 2.7 mM NaHCO_3_, 0.845 mM Na_2_HPO_4_, 1.98 mM CaCl_2_). After a 30 min digestion in Trypsin/EDTA (ethylenediaminetetraacetic acid) and three to four washes with serum medium (2% FCS solution in DMEM), the hippocampi were homogenized and then plated on poly-L-lysine-coated coverslips at a 70 000 cells/cm^2^ density and kept in Neurobasal medium containing 0.25% glutamine, 2% B27 supplement, and 10% 10× N2 supplement. The cultures were incubated at 37 °C and 5% CO_2_.

### Organotypic hippocampal slice cultures

Organotypic hippocampal slice cultures were prepared from P3 Wistar rats. The hippocampi were extracted from the brains and placed in a petri dish containing slicing medium (HBSS (Hanks’ Balanced Salt Solution) (Invitrogen) containing 25 mM HEPES(4-(2-hydroxyethyl)-1-piperazineethanesulfonic acid)). They were then individually cut into 300 μm slices using a tissue chopper. The intact slices were selected and placed on PVDF (polyvinylidene difluoride) cell culture inserts (Millipore) and cultivated in culture medium (containing 50% MEM (Minimum Essential Medium Eagle) with Glutamax-1 (Invitrogen), 24% HBSS (Invitrogen), 36 mM D-glucose, 25% heat-inactivated horse serum (Invitrogen), 1% penicillin-streptomycin, and 0.06% nystatin). For quantitative analysis, only Bassoon puncta that colocalized with Synaptopyhsin were selected.

### Transfection

Neurons were transfected on day in vitro 2 (DIV2) or DIV4 using the calcium phosphate transfection method as described by Dresbach et al. [61]. In short, the original medium was first substituted with OptiMem medium; a mixture containing the plasmid, sterile distilled water, CaCl_2_, and the transfection buffer (274 mM NaCl, 10 mM KCl, 1.4 mM Na_2_HPO_4_, 15 mM glucose, 42 mM HEPES, pH 7.06) was prepared and incubated for 20 min before being added to the cell medium. The cells were incubated for 60 min and then washed with b=neurobasal medium. In the end, the original medium was restored. The following constructs were overexpressed: a farnesylated GFP; an mOrange construct; an NL1 construct containing a GFP tag inserted into the intracellular domain [62]; a newly generated construct containing the NL1 sequence, an internal ribosomal entry site (IRES), and mOrange; and a newly generated construct containing a mutated NL1 sequence (NL1-32) that does not bind to neurexins [34], an IRES, and mOrange. All constructs were inserted into a *pN1* vector.

### Application of BDNF, TrkB-Fc scavenger, and latrunculin A

BDNF (R&D Systems, cat. No. 248-BDB-050) was added at a concentration of 0.1 μg/mL to non-transfected neurons 22 h before fixation, and 1% BSA (bovine serum albumin) dissolved in sterile PBS (phosphate-buffered saline, used to reconstitute BDNF) was added to the cell medium in the control condition. TrkB-Fc (R&D Systems, cat. No. 688-TK-100) dissolved in sterile PBS was added at a final concentration of 4 μg/mL to inhibit the TrkB receptor on DIV3 (12 h after transfection). Latrunculin A (Sigma-Aldrich, cat. No. L5163) dissolved in DMSO (dimethyl sulfoxide) was applied to the medium 6 h prior to fixation at a concentration of 2.5 μM [29]. To visualize F-actin, TRITC (tetramethylrhodamine)-labeled phalloidin was used (1:6000). Anisomycin (Tocris Bioscience, cat. No. 1290) dissolved in DMSO was applied at a final concentration of 10 μM to the medium 6 h prior to fixation.

### Synaptotagmin-1 antibody uptake

A mouse monoclonal antibody directed against the lumenal domain of synaptotagmin-1 (Synaptic Systems, RRID:AB_993036) was diluted (1:200) in depolarization buffer (64 mM NaCl, 70 mM KCl, 2 mM CaCl_2_, 1 mM MgCl_2_, 20 mM HEPES, 30 mM glucose, pH 7.4) to reach a final dilution of 1:600 in the cell medium. The neurons were then incubated for 5 min and subsequently washed three times with neurobasal medium.

### Recombinant lentivirus production and infection

The production of the lentivirus was performed in HEK293T cells (5 × 10^6^ cells in a 10 cm cell culture dish). The cells were co-transfected in serum-free DMEM with 10 μg of the lentiviral expression vector (pUbc–Cre–IRES–GFP) in conjunction with the 7.5 μg of delta 8.9 vector and 5 μg of VSV-G using polyethylenimine (PEI, Sigma-Aldrich). The transfection solution was incubated for 30 min at room temperature and then added dropwise to the cells. Between 4 and 6 h after the transfection, the culture medium was replaced by fresh DMEM containing 10% FCS. Lentivirus was harvested from the cell culture supernatant 48–72 h after transfection and immediately stored at − 70 °C. To excise the *bdnf* gene, primary hippocampal neurons from mice carrying two floxed *bdnf* alleles were transduced with the *cre*-recombinase-expressing lentivirus. The lentivirus-containing medium was added dropwise to the primary neurons in a 1:5 dilution. The transduction was performed 4 h after preparation of the primary hippocampal cultures.

### Immunofluorescence and microscopy

Neurons were fixed with 4% PFA (paraformaldehyde) in K^+^-free PBS for 20 min. Afterwards, they were permeabilized using PBS with 0.3% Triton X-100, 2% BSA, 10% FCS, and 5% glucose.

The primary antibodies were the following: mouse anti-bassoon (Enzo Life Sciences, RRID:AB_2313990) 1:1000, guinea pig anti-bassoon (Synaptic Systems, RRID:AB_2290619), rabbit anti-synaptophysin (Synaptic Systems, RRID:AB_2810218) 1:500, mouse anti-synaptotagmin-1 (Synaptic Systems, RRID:AB_993036) 1:200, chicken anti-MAP2 (microtubule-associated protein 2) (Synaptic Systems, RRID:AB_2619881) 1:1000, rabbit anti-GFP (Abcam, RRID:AB_305564) and knockout-validated mouse anti-BDNF [63] (mouse monoclonal antibody developed by Y.-A. Barde, obtained from the Developmental Studies Hybridoma Bank, created by the NICHD of the NIH and maintained at the University of Iowa, Department of Biology, Iowa City, IA 52242, USA; RRID:AB_2617199) 1:100. They were diluted in PBS with 0.3% Triton X-100, 2% BSA, 10% FCS, and 5% glucose.

The secondary antibodies were the following: anti-mouse Alexa 488 1:1000 (Jackson ImmunoResearch Labs, RRID:AB_2341099), anti-mouse Alexa 647 1:1000 (Jackson ImmunoResearch Labs, RRID:AB_2340866), anti-mouse STAR 580 (Abberior, RRID:AB_2620153), anti-chicken Cy3 1:500 (Jackson ImmunoResearch Labs, RRID:AB_2340363), anti-chicken Alexa 488 (Jackson ImmunoResearch Labs, RRID:AB_2337390), anti-guinea pig Alexa 647 1:1000 (Jackson ImmunoResearch Labs, RRID:AB_2340476), anti-guinea pig STAR 635P (Abberior, cat. No. ST635P-1006), anti-rabbit Alexa 488 1:1000 (Jackson ImmunoResearch Labs, RRID:AB_2338046), anti-rabbit STAR 580 (Abberior, RRID:AB_2810981), and anti-rabbit STAR 635P (Abberior, cat. No. ST635P-1002). They were diluted in PBS with 0.3% Triton X-100, 2% BSA, and 5% glucose.

Epifluorescence microscopy images were acquired using a CoolSNAP HQ2 CCD camera (Photometrics) attached to a Zeiss AxioObserver Z1 with a 40× magnification or a Hamamatsu Orca Flash V2 attached to a Zeiss AxioImager M2. Exposure time for the images with AZ and vesicle labeling was kept consistent for each experiment.

STED microscopy images were acquired using a setup by Abberior Instruments with a 100× magnification. The images were acquired at a pixel size of 10 nm, with a pinhole set at 1.0 AU, and a 5 μs dwell time. The power of the STED 775 laser was set to 30% for the 635 nm channel and to 45% for the 580 nm channel. The STED resolution was 60 nm for the 635 excitation and 90 nm for the 580 nm excitation.

### Image analysis

900 × 900 pixel selections (the average size of seven cell somas) of each image were made with Adobe Photoshop. Synaptic puncta were counted using OpenView (created by Noam E Ziv, Technion, Haifa). Synaptic puncta were selected automatically after setting a threshold and keeping it constant for all images. For the analysis in Fig. 4, only bassoon puncta colocalizing with synaptophysin were counted. Dendritic length was measured in MetaMorph (Molecular Devices) or ImageJ. Synaptotagmin-1 intensity was measured using OpenView and ImageJ. The threshold was set in a way that all visible puncta in a neuron were selected, and it was kept constant throughout a set. Statistical analysis involved a two-tailed Student’s *t* test and a two-way ANOVA that was performed using Prism (GraphPad Software).

### Electrophysiology

Whole-cell voltage clamp recordings of cultured hippocampal neurons were performed at 27 ± 0.1 °C using a HEKA EPC-10 amplifier and the PatchMaster software (HEKA). Data were sampled at 50 kHz and Bessel filtered at 2.9 kHz. Patch pipettes were freshly pulled from borosilicate glass capillaries and had open-tip resistances between 3.5 and 5 MΩ, whereas patched cells had a series resistance under 15 MΩ. Pipettes were filled with solution containing 123 mM Cs-gluconate, 8 mM NaCl, 10 mM HEPES, 10 mM glucose, 10 mM BAPTA (1,2-bis(*o*-aminophenoxy)ethane-*N*,*N*,*N′*,*N′*-tetraacetic acid), 5 mM ATP (adenosine triphosphate)-Mg, and 0.4 mM GTP (guanosine triphosphate)-Na; 300 mOsm/kg and pH 7.2. The extracellular solution contained 119 mM NaCl, 2.5 mM KCl, 26 mM NaHCO_3_, 1 mM NaH_2_PO_4_, 4 mM CaCl_2_, 4 mM MgSO_4_, 10 mM glucose, and 0.001 mM tetrodotoxin (TTX); 300 mOsm/kg. The extracellular solution was constantly gassed with carbogen (95% O_2_, 5% CO_2_) and had a flow rate of 2 mL/min. Recordings of BDNF-treated neurons were performed 24 h after application of BDNF (0.1 μg/mL). Recordings were made for 2 min before application of PMA. PMA (1 μM) was applied for 2 min, and cells were further recorded for 1–2 min. Miniature postsynaptic current (PSC) events were detected using the MiniAnalysis software (Synaptosoft) using a threshold of 9 pA. Statistical significance was determined by a Wilcoxon matched-pairs signed ranked test where n.s. indicates a non-significant change and ** indicates *P*< 0.01.

## Supplementary Information

**Additional file 1: Fig. S1.** Separation of the three fluorescent channels for the panels in Figure 1 A. Scale bar is 10 μm.

**Additional file 2: Fig. S2.** Separation of the three fluorescent channels for the panels in Figure 5 E. Scale bar is 10 μm.

**Additional file 3: Fig. S3.** (A) Separation of the three fluorescent channels for the panels in Figure 6 E. (B) Separation of the three fluorescent channels for the panels in Figure 6 G. Scale bar is 10 μm.

**Additional file 4: Fig. S4.** Similar effects of NL1 and a neurexin-binding-deficient NL1 mutant on structural andfunctional presynaptic maturation. (A) DIV5 cultured hippocampal neurons transfected with NL1–IRES–mOrange (NL1) or mutated NL1–IRES–mOrange (NL1 mutant), treated with LatA or DMSO, and immunostained for bassoon (magenta). (B) Quantification of the number of bassoon puncta per 10 μm dendrite for the conditions indicated in panel A. Mean + SEM; *N* = 3 experiments, *n* = 10 cells per experiment; two-way ANOVA with post-hoc Sidak tests: *p*> 0.05 for NL1 control vs. NL1 + LatA and for NL1 mutant control vs. NL1 mutant + LatA (C) The cultures were stimulated in the presence of antibodies directed against the lumenal domain of synaptotagmin-1 (Syt1) to label recycling synaptic vesicles (magenta). (D) Quantification of the fluorescence intensity of the Syt1 label for the conditions indicated in panel C. Mean + SEM; *N* = 3 experiments, *n* = 10 cells per experiment and condition; Student’s t test: *p*> 0.05. Scale bar is 10 μm.

**Additional file 5: Fig. S5.** Similar effects of NL1 and a neurexin-binding-deficient NL1 mutant on structural presynaptic maturation in NL1-KO cultures. (A) DIV5 cultured cortical WT and NL1-KO neurons transfected with NL1–IRES–mOrange (NL1) or mutated NL1–IRES–mOrange (NL1 mutant), treated with LatA or DMSO, and immunostained for bassoon (magenta). (B) Quantification of the number of bassoon puncta per 10 μm dendrite for the conditions indicated in panel A. Mean + SEM; *N* = 3 experiments, *n* = 10 cells per experiment and condition; two-way ANOVA with post-hoc Sidak tests: *p*> 0.05 for all comparisons. Scale bar is 10 μm.

**Additional file 6: Fig. S6.** (A) Separation of the three fluorescent channels for the panels in Figure 8 A. (B) Separation of the fluorescent channels for the panels in Figure 8 C. Scale bar is 10 μm.

## Data Availability

All data generated or analyzed during this study are included in this published article and its supplementary information files.

## Abbreviations

ASD: Autism spectrum disorders
ATP: Adenosine triphosphate
AZ: Active zone
BAPTA: 1,2-bis(*o*-aminophenoxy)ethane-*N*,*N*,*N′*,*N′*-tetraacetic acid
BDNF: Brain-derived neurotrophic factor
BSA: Bovine serum albumin
CaMKII: Ca^2+^/calmodulin-dependent protein kinase II
DIV: Day in vitro
DMEM: Dulbecco’s minimal essential media
DMSO: Dimethyl sulfoxide
EDTA: Ethylenediaminetetraacetic acid
EGFP: Enhanced green fluorescent protein
EPSC: Excitatory postsynaptic current
FCS: Fetal calf serum
GABA: *Gamma-*aminobutyric acid
GBSS: Gey’s Balanced Salt Solution
GFP, GFP-F: (Farnesylated) Green fluorescent protein
GTP: Guanosine triphosphate
HBSS: Hanks’ Balanced Salt Solution
HEPES: 4-(2-hydroxyethyl)-1-piperazineethanesulfonic acid
IRES: Internal ribosomal entry site
KO: Knockout
LatA: Latrunculin A
LIMK1: LIM domain kinase 1
MAP 2: Microtubule-associated protein 2
MEM: Minimum Essential Media
NL: Neuroligin
NMDA: N-Methyl-D-aspartate
PBS: Phosphate-buffered saline
PEI: Polyethylenimine
PFA: Paraformaldehyde
PMA: Phorbol 12-myristate 13-acetate
PSC: Postsynaptic current
PVDF: Polyvinylidene difluoride
STED: Stimulated emission depletion
SV: Synaptic vesicle
Syt1: Synaptotagmin-1
TRITC: Tetramethylrhodamine
TrkB, TrkB-Fc: Tyrosine receptor kinase B (Fc fragment)
WT: Wild type
