# Supplementary figures and images for: Neuroligin-1 mediates presynaptic maturation through brain-derived neurotrophic factor signaling
Source: BMC Biol. 2021 Sep 27;19:215. doi: 10.1186/s12915-021-01145-7 (PMC8474808; doi:10.1186/s12915-021-01145-7)

Additional File 1: Fig. S1

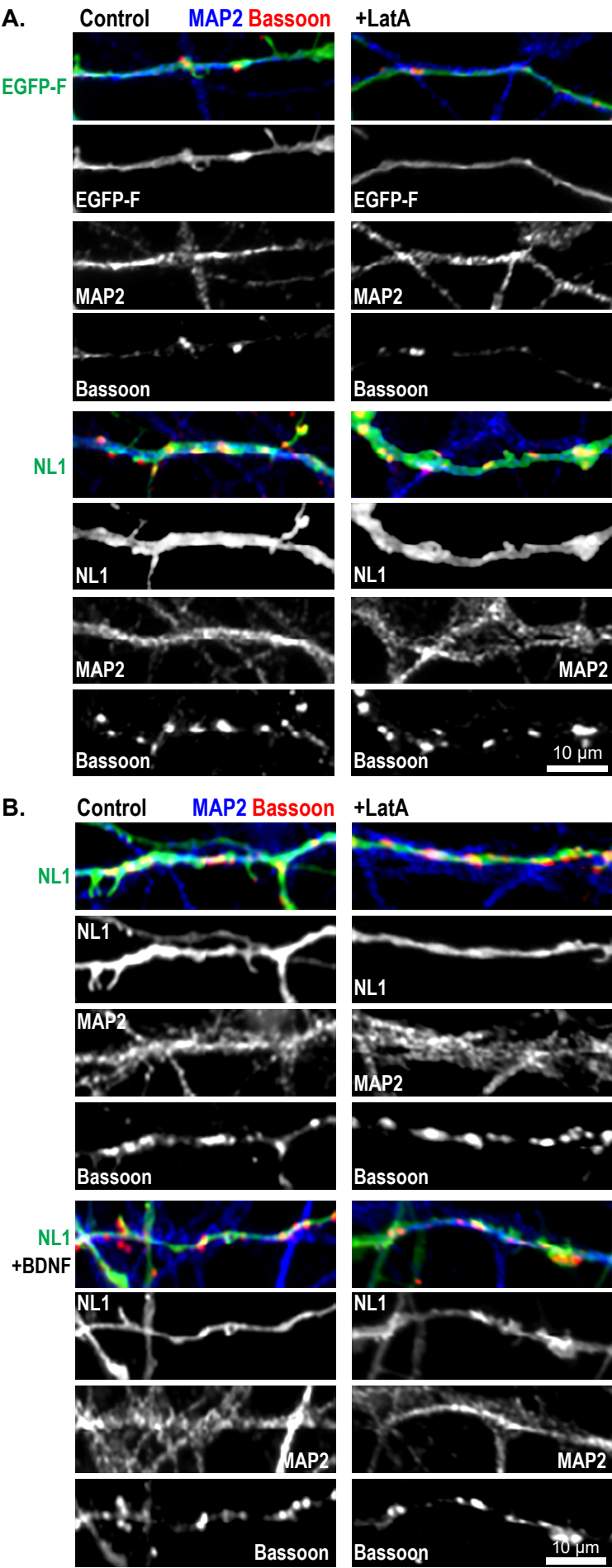

Supplement: Supplementary file 1 — Additional file 1: Fig. S1. Separation of the three fluorescent channels for the panels in Figure 1 A. Scale bar is 10 μm. [file 12915_2021_1145_MOESM1_ESM.pdf]

Additional File 2: Fig. S2

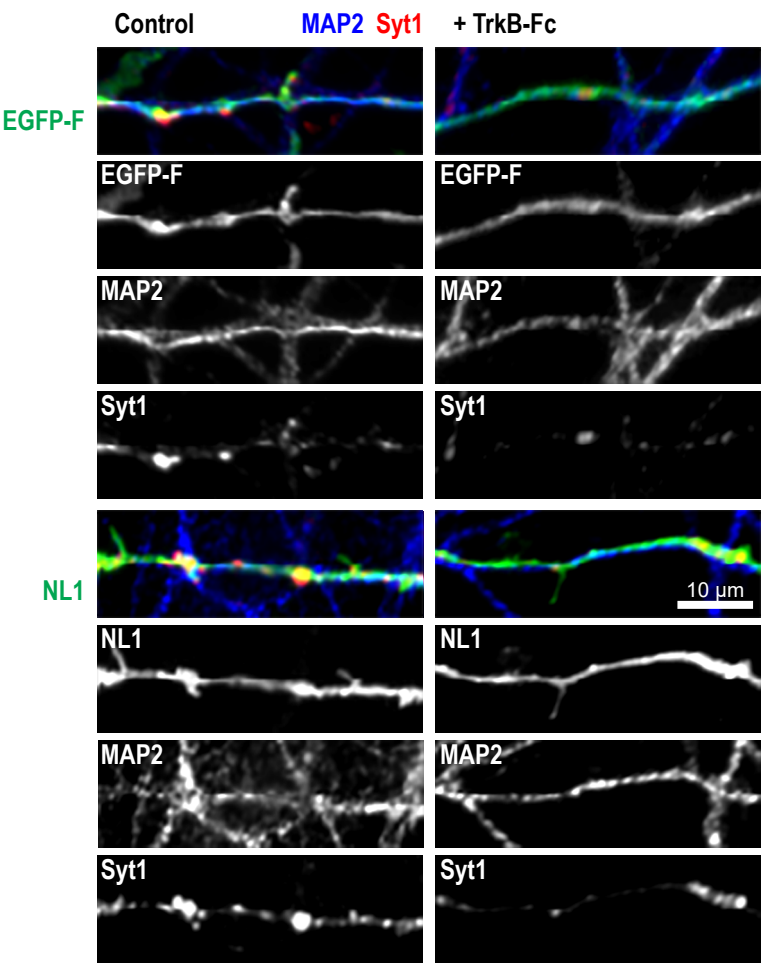

Supplement: Supplementary file 2 — Additional file 2: Fig. S2. Separation of the three fluorescent channels for the panels in Figure 5 E. Scale bar is 10 μm. [file 12915_2021_1145_MOESM2_ESM.pdf]

# Additional File 3: Fig. S3

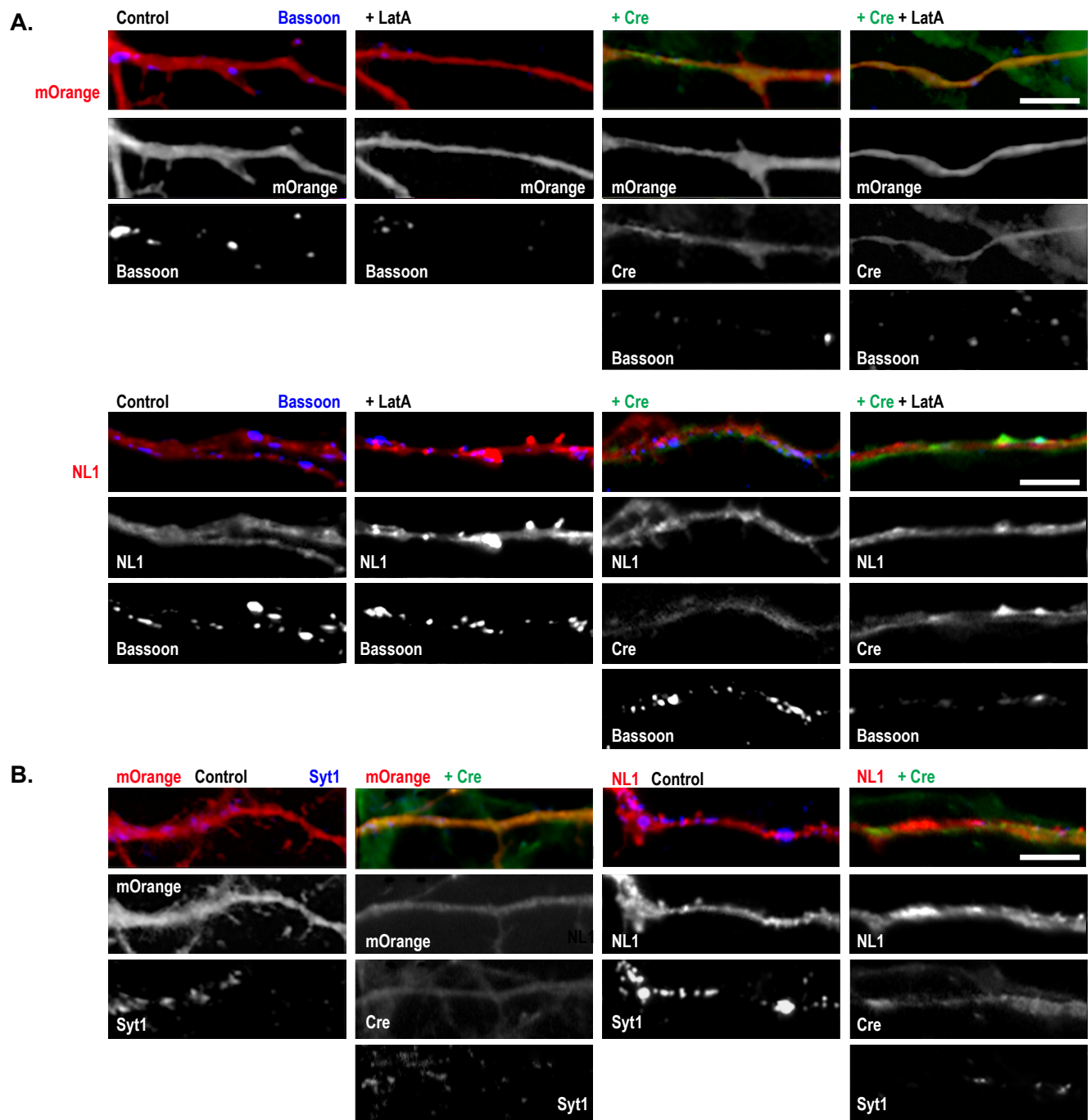

Supplement: Supplementary file 3 — Additional file 3: Fig. S3. (A) Separation of the three fluorescent channels for the panels in Figure 6 E. (B) Separation of the three fluorescent channels for the panels in Figure 6 G. Scale bar is 10 μm. [file 12915_2021_1145_MOESM3_ESM.pdf]

Additional File 4: Fig. S4

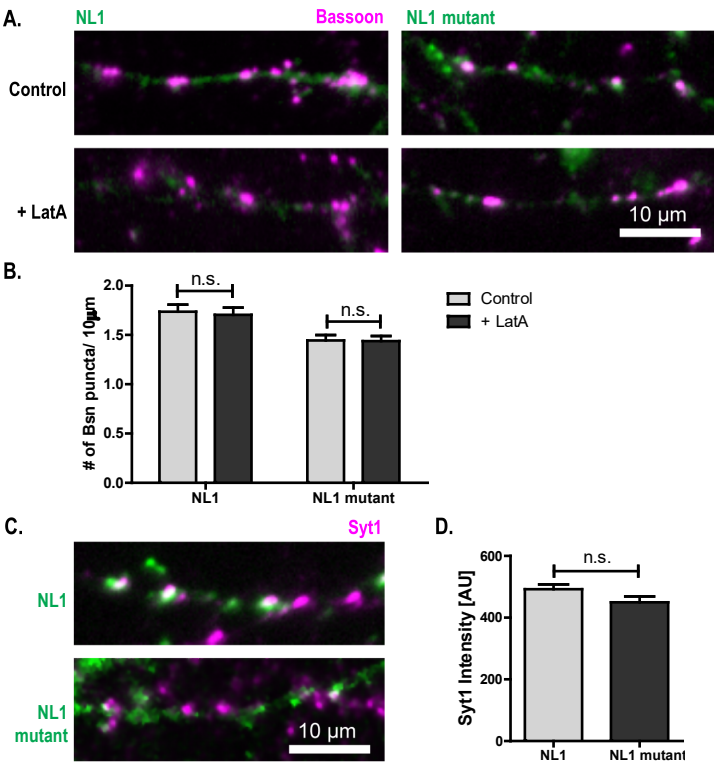

Supplement: Supplementary file 4 — Additional file 4: Fig. S4. Similar effects of NL1 and a neurexin-binding-deficient NL1 mutant on structural andfunctional presynaptic maturation. (A) DIV5 cultured hippocampal neurons transfected with NL1–IRES–mOrange (NL1) or mutated NL1–IRES–mOrange (NL1 mutant), treated with LatA or DMSO, and immunostained for bassoon (magenta). (B) Quantification of the number of bassoon puncta per 10 μm dendrite for the conditions indicated in panel A. Mean + SEM; N = 3 experiments, n = 10 cells per experiment; two-way ANOVA with post-hoc Sidak tests: p> 0.05 for NL1 control vs. NL1 + LatA and for NL1 mutant control vs. NL1 mutant + LatA (C) The cultures were stimulated in the presence of antibodies directed against the lumenal domain of synaptotagmin-1 (Syt1) to label recycling synaptic vesicles (magenta). (D) Quantification of the fluorescence intensity of the Syt1 label for the conditions indicated in panel C. Mean + SEM; N = 3 experiments, n = 10 cells per experiment and condition; Student’s t test: p> 0.05. Scale bar is 10 μm. [file 12915_2021_1145_MOESM4_ESM.pdf]

Additional File 5: Fig. S5

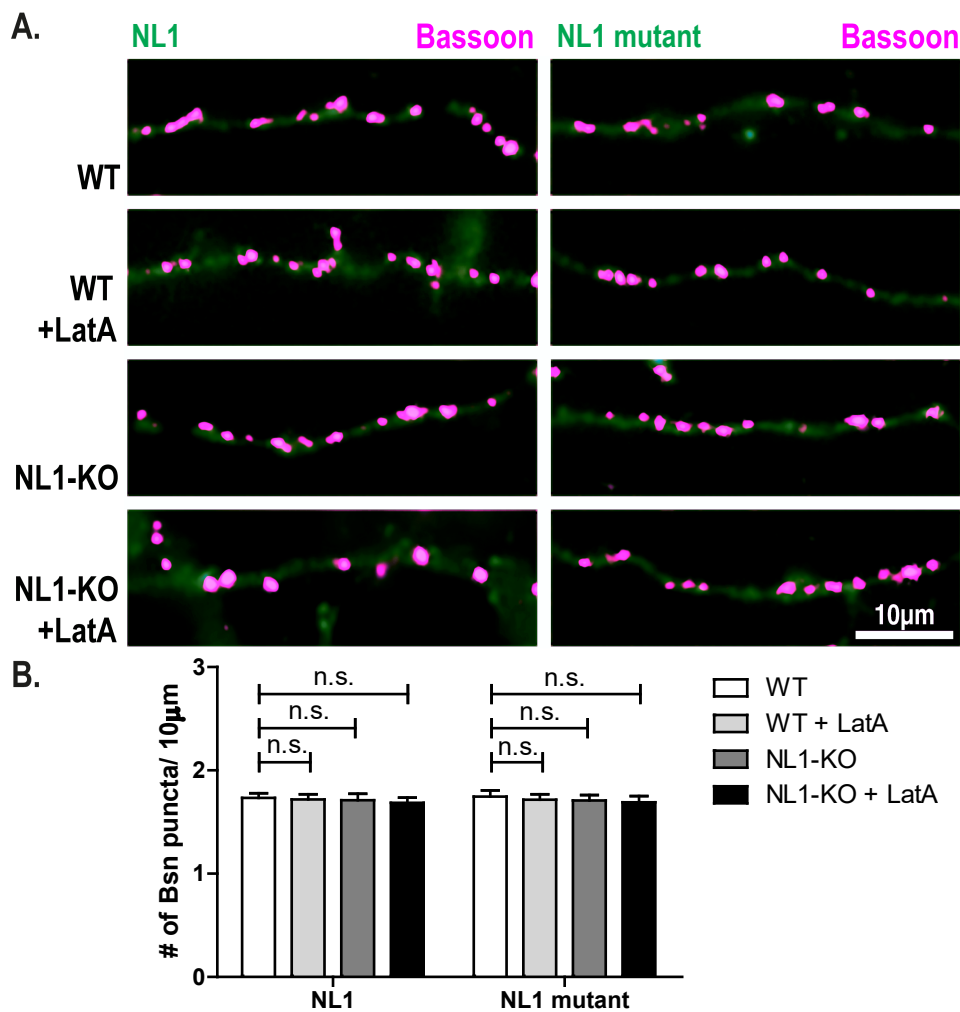

Supplement: Supplementary file 5 — Additional file 5: Fig. S5. Similar effects of NL1 and a neurexin-binding-deficient NL1 mutant on structural presynaptic maturation in NL1-KO cultures. (A) DIV5 cultured cortical WT and NL1-KO neurons transfected with NL1–IRES–mOrange (NL1) or mutated NL1–IRES–mOrange (NL1 mutant), treated with LatA or DMSO, and immunostained for bassoon (magenta). (B) Quantification of the number of bassoon puncta per 10 μm dendrite for the conditions indicated in panel A. Mean + SEM; N = 3 experiments, n = 10 cells per experiment and condition; two-way ANOVA with post-hoc Sidak tests: p> 0.05 for all comparisons. Scale bar is 10 μm. [file 12915_2021_1145_MOESM5_ESM.pdf]

Additional File 6: Fig. S6

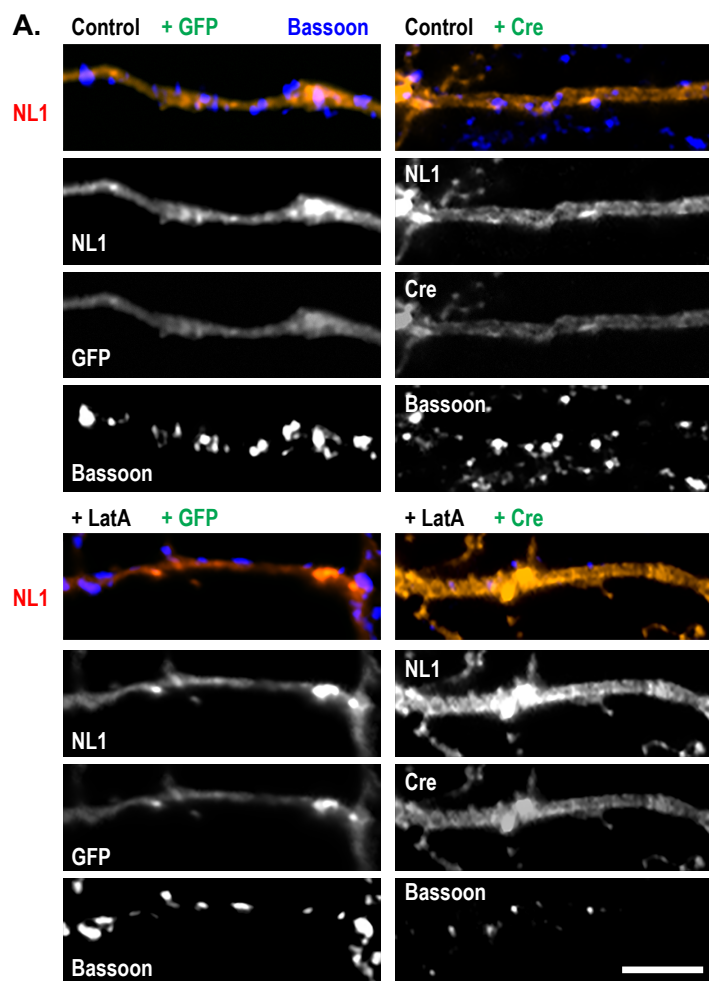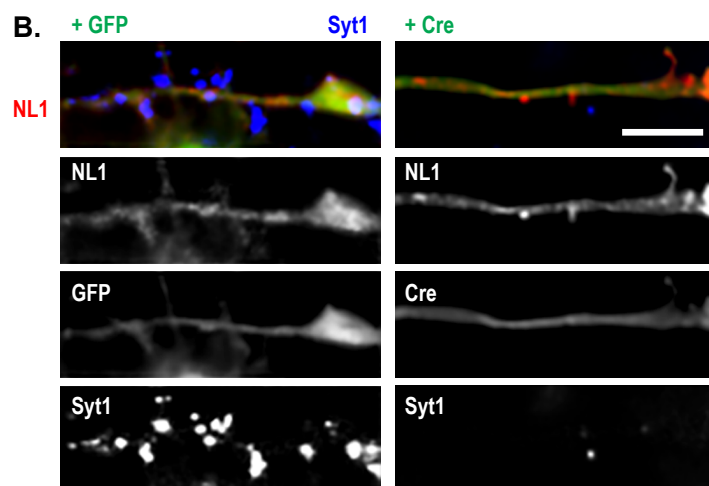

Supplement: Supplementary file 6 — Additional file 6: Fig. S6. (A) Separation of the three fluorescent channels for the panels in Figure 8 A. (B) Separation of the fluorescent channels for the panels in Figure 8 C. Scale bar is 10 μm. [file 12915_2021_1145_MOESM6_ESM.pdf]
